# Associations Between Anthropogenic Factors, Meteorological Factors, and Cause‐Specific Emergency Department Admissions

**DOI:** 10.1029/2024GH001061

**Published:** 2024-09-04

**Authors:** Pranav Tewari, Baihui Xu, Ma Pei, Kelvin Bryan Tan, John Abisheganaden, Steve Hung‐Lam Yim, Borame Lee Dickens, Jue Tao Lim

**Affiliations:** ^1^ Lee Kong Chian School of Medicine Nanyang Technological University Singapore Singapore; ^2^ Saw Swee Hock School of Public Health National University of Singapore Singapore Singapore; ^3^ Ministry of Health Singapore Singapore; ^4^ Tan Tock Seng Hospital Singapore Singapore; ^5^ Asian School of the Environment Nanyang Technological University Singapore Singapore

**Keywords:** non‐linear associations, generalized additive models, population attributable fraction, Singapore, emergency department admissions

## Abstract

Unpredictable emergency department (ED) admissions challenge healthcare systems, causing resource allocation inefficiencies. This study analyses associations between air pollutants, meteorological factors, and 2,655,861 cause‐specific ED admissions from 2014 to 2018 across 12 categories. Generalized additive models were used to assess non‐linear associations for each exposure, yielding Incidence Rate Ratios (IRR), while the population attributable fraction (PAF) calculated each exposure's contribution to cause‐specific ED admissions. IRRs revealed increased risks of ED admissions for respiratory infections (IRR: 1.06, 95% CI: 1.01–1.11) and infectious and parasitic diseases (IRR: 1.09, 95% CI: 1.03–1.15) during increased rainfall (13.21–16.97 mm). Wind speeds >12.73 km/hr corresponded to increased risks of ED admissions for respiratory infections (IRR: 1.12, 95% CI: 1.03–1.21) and oral diseases (IRR: 1.58, 95% CI: 1.31–1.91). Higher concentrations of air pollutants were associated with elevated risks of cardiovascular disease (IRR: 1.16, 95% CI: 1.05–1.27 for PM_10_) and respiratory infection‐related ED admissions (IRR: 2.78, 95% CI: 1.69–4.56 for CO). Wind speeds >12.5 km/hr were predicted to contribute toward 10% of respiratory infection ED admissions, while mean temperatures >28°C corresponded to increases in the PAF up to 5% for genitourinary disorders and digestive diseases. PM_10_ concentrations >60 μg/m^3^ were highly attributable toward cardiovascular disease (PAF: 10%), digestive disease (PAF: 15%) and musculoskeletal disease (PAF: 10%) ED admissions. CO concentrations >0.6 ppm were highly attributable to respiratory infections (PAF: 20%) and diabetes mellitus (PAF: 20%) ED admissions. This study underscores protective effects of meteorological variables and deleterious impacts of air pollutant exposures across the ED admission categories considered.

## Key Points

- High total daily rainfall was negatively associated with emergency department (ED) admissions, while mean temperature was positively associated with ED admissions
- Increased wind speed contributed disproportionately to increased ED admissions due to respiratory infections
- Increased ambient air pollutant concentrations had the greatest impact on the categories belonging to the cardiorespiratory system disorders

## 1 Background

The ability to accurately manage the influx of patients seeking emergency care is critical for healthcare systems (Y. Sun et al., 2009). The unpredictability and variability of emergency department (ED) admissions load pose significant challenges for healthcare systems, as it can lead to a strain in resources. Prior studies have revealed that the poor management of ED admissions and overcrowding often has adverse consequences on patient outcomes (Barak‐Corren et al., 2021; Harrou et al., 2015, 2022; Hurwitz et al., 2014). Understanding risk factors leading to higher ED admission loads provide an opportunity to anticipate surges, identify potential trends, and allocate resources in a pre‐emptive manner, thereby enhancing the responsiveness and preparedness of healthcare systems.

Several studies have explored the effects of meteorological factors and ambient air pollutants on ED admissions (Altuntas et al., 2023; Wang et al., 2020; Zhou et al., 2021), particularly during the coronavirus disease 2019 pandemic. Among the multitude of studies that have tested the influence of weather‐related variables on ED admissions, temperature has emerged as the most frequently examined variable. Wang et al. (2020) reported that higher temperature was a significant risk factor resulting in increases in ED admissions, although the associations varied across the different city and county regions in China. Higher maximum temperatures and the occurrence of heat waves have also been linked to increased ED visits (Duwalage et al., 2020) as well as mortality rates, especially among elderly (Schaffer et al., 2012). Similarly, other weather‐related variables, such as precipitation and wind have also been documented to have a significant influence on ED admissions (Calegari et al., 2016).

Air pollution is a complex environmental concern that has garnered substantial attention due to its potential health implications. With increasing urbanization and industrialization, the impact of air pollutant concentration on public health has become a pressing matter. High concentrations of ambient air pollutants such as particulate matter, NO_2_, SO_2_, and O_3_ have been associated with adverse impacts on the respiratory system, especially in relation to asthma ED visits (Anenberg et al., 2018; Bi et al., 2023). Furthermore, fine particles have also been documented to have impacts on cardiovascular diseases (Kim et al., 2012), suggesting that the impact of air pollutants may not be limited to the respiratory system alone.

However, the aforementioned studies often consider ED admissions regardless of category, or focus on only one specific category, despite the evidence that these factors have distinct effects across multiple physiological systems (Fontaine et al., 2018; Iwase et al., 2002; Pryor et al., 2022). Moreover, the models presented in literature often incorporate only either meteorological variables or air pollutant variables and not both, even though air pollution remains an inevitable feature of urban environments. The combined effect of meteorological and ambient air pollutant variables across multiple ED admission categories remains an unexplored aspect of ED modeling, with the potential to yield insights that can aid in understanding the complex interplay between anthropogenic factors, meteorological factors and cause‐specific ED admissions.

Therefore, the primary objective of our study is to understand and quantify the effects of common meteorological variables and air pollutants which are associated with cause‐specific ED admissions in Singapore. First, we harmonized ED admissions with comprehensively recorded daily measurements of meteorological variables and ambient air pollutant levels in the study setting. We used generalized additive models (GAM) to delineate potential non‐linear associations between the ED admissions and variables of interest. The associations were studied by converting the risks of each variable of interest into the incidence rate ratio scale for interpretation. Moreover, we computed the population attributable fraction (PAF) for each variable to quantify the contribution of each exposure on ED admissions.

## 2 Data and Methods

### 2.1 ED Admissions Data

Cause‐specific ED admissions across all public hospitals in Singapore between EW1 2014 to EW 52 2018 were obtained from the Ministry of Health, Singapore. Daily admissions across 20 categories were obtained and narrowed down to 12, after removing categories with missing data or changes in classification of ED admission. For analysis and simpler visualization of results, the 12 admission categories were grouped together based on the similarity of the underlying physiological systems for each condition in the admission category, as presented in Table 1.

**Table 1:** Grouping of Emergency Department Admission Categories

Grouping	Category
Cardiorespiratory system disorders	Cardiovascular disease
	Chronic respiratory disease
	Respiratory infection
	Infectious and parasitic disease
Metabolic and digestive system disorders	Diabetes mellitus
	Digestive disease
	Endocrine disorders
	Genitourinary disorders
Others	Musculoskeletal disease
	Neurological and sense disorders
	Oral diseases
	Skin diseases

### 2.2 Meteorological Data

Well‐established variables which are known to influence ED admissions were collected. These included daily mean temperature, total daily rainfall and wind speed, which were obtained from a total of 21 weather stations installed by the National Environment Agency. We created daily complete raster maps through inverse distance weighting interpolation, which was carried out using cross validation of leave‐one‐out for the fitting of the inverse distancing power to minimize the error in observation on the raster surface of the test point. These values were aggregated at a weekly level to correspond with the ED admissions data.

### 2.3 Ambient Air Pollutant Data

We obtained daily air pollution data for a range of pollutants over the period of 2014–2018 from the Air Quality Open Data Platform. Estimates are provided for 5 zones covering the North, West, East, Central, and South regions of Singapore, covering PM_10_, SO_2_, and CO concentrations. The estimates within each of the five zones were then averaged to obtain values at a national level. Following which, they were converted from AQI index levels to their respective concentration units using the breakpoints and formulas provided in the technical assistance documentation from the United States Environmental Protection Agency (Aqi‐technical‐assistance‐document‐sept2018.pdf, 2024) for use in the statistical models.

### 2.4 Estimating Non‐Linear Relationships Between Cause‐Specific ED Admissions and Environmental and Anthropogenic Exposures

Generalized additive models (GAMs) were used to uncover potential non‐linear relationships between the cause‐specific ED admissions and the exposures considered in this study. Poisson models were constructed for each admission category, with cause‐specific ED admissions being regressed against the meteorological exposures and ambient air pollutants mentioned above. In each instance, meteorological data and ambient air pollution concentration data for the previous 3 weeks were included as explanatory variables. We also utilized each category's lagged weekly case count data, up to a 3‐week lag, in order to account for the temporal dependence of cause‐specific ED admissions. We utilized the Partial Autocorrelation Function (PACF) to determine the appropriate lag in our modeling strategy (Refer to Figure S2 in Supporting Information S1). As our data spanned 5 years, it limited the incorporation of greater lags beyond 3 weeks. The PACF plots also revealed that considering a 3‐week lag would be sufficient for a bulk of the admission categories considered in this study. As a sensitivity analysis, we considered quasi‐Poisson and negative binomial GAM models, as these distributions account and correct for any detected overdispersion in the outcome variable. We further considered different model formula specifications for each of the specified distributions, where we separately considered models with estimated smooth functions where all explanatory variables were at a 1‐, 2‐, and 3‐week lag (Equation 1). Additionally, we tested models that included estimated smooth functions for both the immediate (current week) effects and the lagged effects.

Let *Y* be the response variable (e.g., cause‐specific ED admissions), $X_{i}={\left(X_{1},\text{…},X_{j}\right)}^{\prime }$ and $Z_{i}={\left(Z_{1},\text{…},Z_{j}\right)}^{\prime }$ be the meteorological and the ambient air pollutant covariates respectively. The GAMs assume that:

(1) $$\log\left(E\left[Y_{t}|X\right]\right)=\beta_{0}+\sum_{n=1}^{l}\sum_{i=1}^{3}f_{i}\left(Y_{t-n}\right)+\sum_{n=1}^{l}\sum_{i=4}^{j}f_{i}\left(X_{i,t-n}\right)+\sum_{n=1}^{l}\sum_{i=j+1}^{k}f_{i}\;\left(Z_{i,t-n}\right)+s\left({\text{Week}}_{t}\right)+s\left({\text{Year}}_{t}\right)$$

where *β*_0_ is a constant, *Y*_*t*−*n*_ represents the autoregressive terms included in the models up to a 3‐week lag, l represents an integer up to the value of three to represent the lagged covariates, and *f*_*i*_, *i* = 1,…,*k* are smooth functions for the respective covariate. Seasonal and long term trends are accounted for by estimating smooth functions for epidemic week, Week_*t*_, and year, Year_*t*_ respectively. For all models, the variables included in the GAMs were smoothed using thin plate regression splines, as they offer a solution to the challenges associated with knot placement and have lower mean squared errors compared to knot‐based splines for regressions (Wood, 2003). As the estimation strategy of generalized cross‐validation tends to under‐smooth the exposure‐response curves versus the true values, we utilized restricted maximum likelihood to estimate the splines in the models. The goodness of fit of the models across the three lags considered was assessed using the Akaike Information Criterion (AIC). The AIC was used as it enables comparison across the models while penalizing model complexity (Akaike, 1973). When evaluating models with the quasi‐Poisson approach, we considered the Root Mean Square Error (RMSE) and Mean Absolute Percentage Error (MAPE) as indicators of best fit instead of the AIC.

### 2.5 Deriving Incidence Rate Ratios

We interpreted and visualized the effects of meteorological and ambient air pollutant exposures on cause‐specific ED admissions by computing the Incidence Rate Ratio (IRR). This ratio quantifies the ratio difference in ED admissions given past values of the exposure of interest. To do this, we initially predicted ED admissions by successively varying the observed value for the exposure of interest across its observed range, while keeping all other exposures fixed at their mean values. The IRR estimates for the predicted ED admissions were then obtained by taking the ratio of the predicted ED admissions at varying exposure levels (numerator) to the predicted ED admissions at the mean exposure level, as per Equation 2.

(2) $$\text{IR}R_{i,k}=\frac{E[\hat{Y}|\;\left(X_{i}=X_{i,k},X_{j}=\bar{X_{j}}\right)]}{E\left[\;\hat{Y}|\;\left(X_{i}=\bar{X_{i}},X_{j}=\bar{X_{j}}\right)\right]},$$

where $\hat{Y}$ is the estimated ED admissions, *X*_*i*_ is the exposure of interest, *X*_*i*,*k*_ is the *k*th quantile of the exposure *i*, and *X*_*j*_ is the set of remaining exposures. Thus, the IRR can be expressed as a factor increase or decrease in ED admissions given a value of an exposure of interest, as compared to the ED admissions at the mean value of the exposure of interest, while holding all other exposures at their mean values. IRR estimates and their corresponding confidence intervals were generated to produce the exposure response curves of the relationship between the exposures and ED admissions for each admission category. As IRRs are a quantity that express a ratio difference, IRRs greater than one indicate a positive association, while IRRs less than one indicate a negative association.

### 2.6 Population Attributable Fraction

To quantify the contributive burden of each exposure of interest on cause‐specific ED admissions, we further computed the PAF. Attributable fractions are measures of association between cause specific ED admissions and a specific exposure that attempt to assess the public health impact of that exposure (Lin & Chen, 2019). The PAF estimates the proportion of disease incidence in a population that can be attributed to a specific exposure. The PAF was calculated from the models that provided the best fit to the data using the following steps:

1. The exposure of interest was “removed” by classifying all individuals as unexposed. This was implemented by setting all values of the exposure of interest to be zero for each individual, which effectively removes the smooth function of the specific exposure from the terms that contribute to predicted ED case counts.
2. The re‐classified data from step 1 is supplied to the previously estimated models to estimate ED admissions, which represents the expected number of ED admissions if the exposure of interest was removed from the population.
3. The PAF was calculated for every datapoint, across all admission categories, using Equation 3.

(3) $$\text{PAF}=\frac{\text{Observed}\;\text{Counts}\;-\;\text{Expected}\;\text{Counts}\;\text{from}\;\text{Step}\;2}{\text{Observed}\;\text{Counts}}\times 100\%$$

In order to visualize and interpret the PAF values, the admission categories were split into the three groups as shown above in Table 1. Smoothed line graphs across the PAF data points were plotted based on the above division of categories for each of the exposures of interest.

## 3 Results

### 3.1 Descriptive Analysis

Across the study period of EW1 2014 to EW 52 2018, a total of 2,655,861 ED admissions were observed across the 12 admission categories considered in this study. Respiratory infections contributed to the highest number of ED admissions, with a total of 594,254 admissions over the study period, accounting for 22% of the total admissions observed. The total ED admissions, classified by their respective admission category are presented in Supporting Information S1 (Refer to Figure S1). The time series of the ED admissions across all admission categories reveal no obvious or discernible seasonal patterns (Refer to Figure S3 in Supporting Information S1).

This study was carried out in Singapore, located 1° north of the equator, with meteorological variables observed to be reflective of a tropical climate, as shown in Table 2. The mean temperature over the study period was 28.01°C, with little variation in values and a relatively small observed range of 25.01 to 29.99°C. Relatively larger variations were observed in the total daily rainfall (Range: 0.00–21.72 mm) and mean windspeed values (Range: 5.43–15.24 km/hr). Similarly, ambient air pollutant concentrations displayed significant variation from mean values (Ranges: 16.03–117.01 μg/m^3^, 0.78–8.57 ppb, 0.19–1.10 ppm for PM_10_, SO_2_, and CO respectively), although values were generally low and indicative of good air quality.

**Table 2:** Summary Statistics of Cause‐Specific Emergency Department (ED) Admissions, Meteorological Factors, and Ambient Air Pollutant Exposures Between the Study Period of 2014–2018

Statistic	Mean	St. dev.	Min	Pctl (25)	Pctl (75)	Max
Exposures						
Total daily rainfall (mm)	6.04	4.52	0.00	2.54	8.60	21.72
Mean temperature (°C)	28.01	0.86	25.01	27.42	28.57	29.99
Mean windspeed (km/hr)	8.24	1.81	5.43	6.82	9.24	15.24
PM_10_ concentration (μg/m^3^)	29.06	12.37	16.03	22.62	31.29	117.01
SO_2_ concentration (ppb)	3.81	1.83	0.78	2.10	5.11	8.57
CO concentration (ppm)	0.36	0.11	0.19	0.29	0.41	1.10
ED admissions						
Cardiovascular diseases	873.57	65.92	624	841	918	1,075
Chronic respiratory diseases	598.93	72.68	421	552	645	856
Diabetes mellitus	66.33	15.55	26	54	77	108
Digestive diseases	1,161.74	274.48	648	888	1,403	1,698
Endocrine disorders	323.30	59.01	206	274	372	466
Genitourinary disorders	695.89	55.96	565	653	736	835
Infectious and parasitic diseases	1,362.46	222.48	890	1,189	1,539	2,029
Musculoskeletal diseases	1,211.36	108.05	893	1,134	1,299	1,496
Neurological and sense disorders	800.29	51.93	623	769	834	964
Oral diseases	65.76	10.59	39	59	73	105
Respiratory infections	2,276.84	529.57	1,266	1,898	2,590	4,083
Skin diseases	739.26	62.43	550	700	781	879

### 3.2 Model Assessment

We assessed model performance by comparing AIC, RMSE, and MAPE values. The Poisson models incorporating variables up to a 3‐week lag and excluding immediate effects (i.e., exposures with no lag), provided the best fit across all admission categories, except for diabetes mellitus ED admissions (Refer to Table S1 in Supporting Information S1). As the improvement in model fit was marginal (RMSE with Immediate Effect: 8.24, RMSE without Immediate Effect: 8.52), we opted for the model excluding immediate effects for consistency across all categories. Goodness of fit was further ascertained by visual comparison of the observed time series plots and predicted values of the ED case counts obtained from the 3‐week lag model (Refer to Figure S2 in Supporting Information S1). Consequently, we interpreted IRRs, PAFs, and the associations between the ED case accounts and exposures from the Poisson GAM models including only lagged variables up to a 3‐week lag. All statistical analyses were conducted at the 95% confidence level.

### 3.3 Associations Between Exposures and Cause‐Specific ED Admissions

Incidence rate ratios derived from estimated GAMs demonstrate non‐linear associations between cause‐specific ED admissions and the exposures considered in this study, across all admission categories (Refer to Figure S4 in Supporting Information S1).

**Figure 1:**
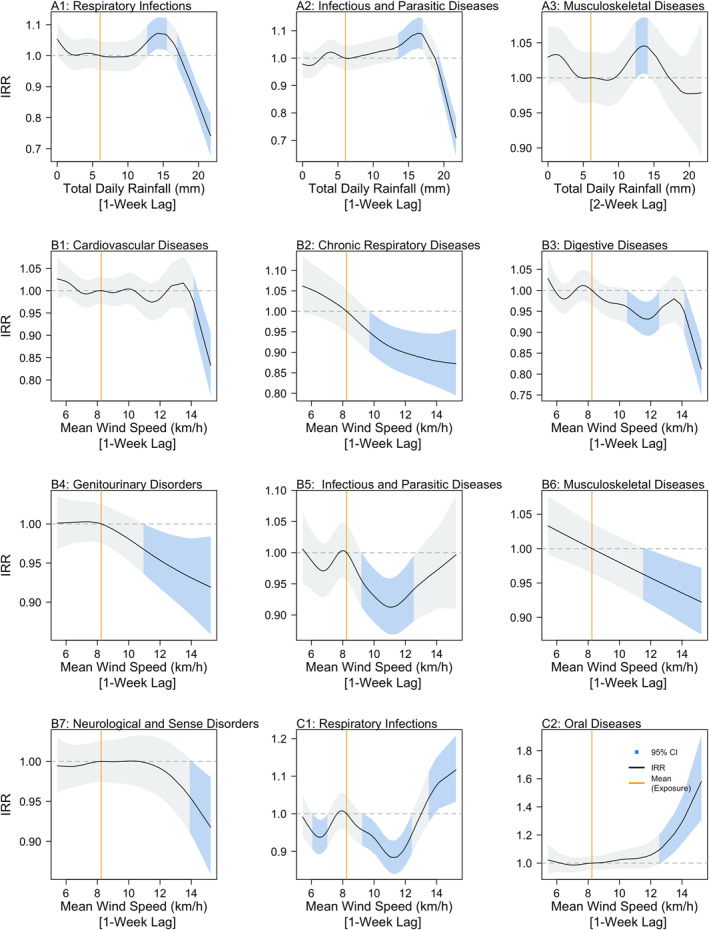
Incidence Rate Ratios of Total Daily Rainfall (a1–a3) and Mean Wind Speed (b1–b7, c1–c2) for cause‐specific emergency department (ED) admissions. Light blue shaded areas represent Incidence Rate Ratios (IRRs) where 95% confidence intervals do not cross 1, while the orange lines represent the mean recorded measurement of the respective exposure, as a reference value. Black lines represent IRR estimates, indicating the factor change in cause‐specific ED admissions across the observed range of the exposure of interest, relative to the mean value of that exposure.

In general, negative associations were predicted between ED admissions and total daily rainfall values above 15 mm for 1‐, 2‐, and 3‐week lags. Exceptions were observed however for admissions due to respiratory infections, infectious and parasitic diseases and musculoskeletal diseases categories, where positive associations were estimated. For the respiratory infection category, an increased risk of ED admission was predicted, ranging from a 6% (IRR: 1.06, 95% CI: 1.01–1.11) to a 7% (IRR: 1.07, 95% CI: 1.02–1.12) increase as total daily rainfall 1‐week prior increased from 13.42 to 15.28 mm (Figure 1a1), when taking mean values as a reference. For the infectious and parasitic disease category, an increased risk of ED admission by 5% (IRR: 1.05, 95% CI: 1.01–1.10) to 9% (IRR: 1.09, 95% CI: 1.03–1.15) was predicted as total daily rainfall 1‐week prior increased from 14.13 to 16.97 mm (Figure 1a2). For the musculoskeletal disease category, an increased risk of ED admissions by 4% (IRR: 1.04, 95% CI: 1.01–1.09) to 5% (IRR: 1.05, 95% CI: 1.01–1.09) was predicted as total daily rainfall 2‐weeks prior increased from 13.21 to 13.54 mm (Figure 1a3). For the remainder of the admission categories, total daily rainfall values below 15 mm were predicted to have non‐significant associations, as the confidence intervals included 1 across the range of specified values. The associations between predicted ED admissions and mean temperature were mixed, with varying associations across the differing lags considered and the different admission categories. However, in general, significant associations between the predicted ED admissions and temperature were more common when considering mean temperature at a 2 to 3‐week lag, indicating an estimated delayed effect of mean temperature on cause‐specific ED admissions (Refer to Figure S4 in Supporting Information S1). Wind speeds above 8.24 km/hr were estimated to be negatively associated with ED admissions across several categories, when considering a 1‐week lag across the following categories: cardiovascular disease, chronic respiratory disease, digestive disease, genitourinary disorders, infectious, and parasitic diseases, musculoskeletal disease, and neurological and sense disorders (Figures 1b1–1b7). Notable exceptions were the respiratory infection and oral diseases categories, where wind speeds above 12 km/hr were estimated to be positively associated with ED admissions. For the respiratory infection category, the risk of ED admissions was predicted to increase by 7% (IRR: 1.07, 95% CI: 1.01–1.14) to 12% (IRR: 1.12, 95% CI: 1.03–1.21) as wind speed increased from 13.89 to 15.53 km/hr (Figure 1c1), while for the oral diseases category, the risk of ED admissions was predicted to increase by 11% (IRR: 1.11, 95% CI: 1.01–1.23) to 58% (IRR: 1.58, 95% CI: 1.31–1.91) as wind speed increased from 12.73 to 15.24 km/hr (Figure 1c2). These indicate positive predicted associations between ED admissions and high wind speeds 1‐week prior for the two admission categories.

**Figure 2:**
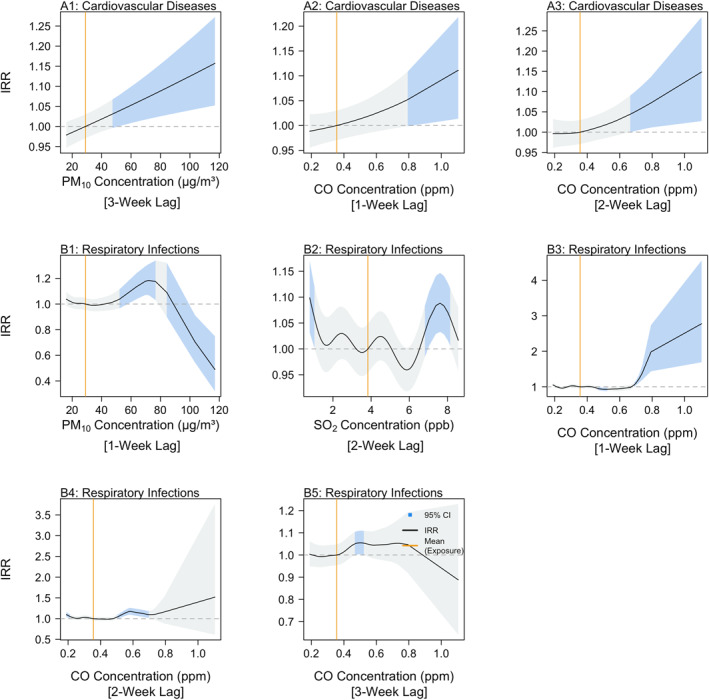
Incidence Rate Ratios of PM_10_ concentration (a1, b1), CO concentration (a2–a3, b3–b5) and SO_2_ concentration (b2) for cause‐specific emergency department (ED) admissions. Light blue shaded areas represent Incidence Rate Ratios (IRRs) where 95% confidence intervals do not cross 1, while the orange lines represent the mean recorded measurement of the respective exposure, as a reference value. Black lines represent IRR estimates, indicating the factor change in cause‐specific ED admissions across the observed range of the exposure of interest, relative to the mean value of that exposure.

Higher ambient air pollutant concentrations demonstrated varying associations with the ED admissions, across the different pollutants, time lags and admission categories considered in this study. In particular, the ED admissions due to cardiovascular disease and respiratory infections were estimated to be the most sensitive to increases in ambient air pollution concentrations, with IRRs being predicted to be greater than 1.10 for select ambient air pollutants. These indicate that the ED admissions for the two categories were predicted to increase by more than 10%, as compared to the expected admissions when the pollutants are held at their mean value. For the cardiovascular disease category, PM_10_ concentrations above 43.59 μg/m^3^ 3 weeks prior were predicted to be positively associated with ED admissions, as the risk of ED admissions was predicted to increase by 2% (IRR: 1.02, 95% CI: 1.00–1.06) to 16% (IRR: 1.16, 95% CI: 1.05–1.27) as PM_10_ concentrations increased from 43.59 to 117.00 μg/m^3^ (Figure 2a1). Similarly, CO concentrations above 0.8 and 0.65 ppm, 1‐ and 2‐weeks prior respectively, were predicted to be positively associated with cardiovascular disease ED admissions. An increase in CO concentrations 1‐week prior from 0.79 to 1.10 ppm (Figure 2a2) corresponded to a predicted increase in ED admissions risk from 5% (IRR: 1.05, 95% CI: 1.00–1.11) to 11% (IRR: 1.11, 95% CI: 1.01–1.22), and an increase in CO concentration 2‐weeks prior from 0.66 to 1.10 ppm corresponded (Figure 2a3) to a predicted increase in risk of ED admissions from 4% (IRR: 1.04, 95% CI: 1.00–1.09) to 15% (IRR: 1.15, 95% CI: 1.03–1.28). For the respiratory infections category, PM_10_ concentrations 1‐week prior between 63.86 and 76.67 μg/m^3^ were positively associated with ED admissions (Figure 2b1), corresponding to a predicted increased risk of ED admissions by 14% (IRR: 1.14, 95% CI: 1.04–1.24) and 18% (IRR: 1.18, 95% CI: 1.03–1.32). This indicates a predicted immediate effect of high PM_10_ concentrations on respiratory infection ED admissions. Similarly, higher SO_2_ concentrations 2 weeks prior were estimated to be positively associated with respiratory infection ED admissions. An increase in SO_2_ concentration 2 weeks prior from 7.05 to 7.82 ppb (Figure 1, b2) corresponded to a predicted increase in ED admission risk by 6% (IRR: 1.06, 95% CI: 1.01–1.11) to 8% (IRR: 1.08, 95% CI: 1.03–1.14). Higher levels of CO concentrations were estimated to drastically increase respiratory infection ED admissions, as increases in CO concentrations 1 week prior from 0.69 to 1.10 ppm (Figure 2b3) corresponded to a predicted increase in ED admissions risk by 8% (IRR: 1.08, 95% CI: 1.01–1.14) to 178% (IRR: 2.78, 95% CI: 1.69–4.56), while increases in CO concentrations 2 weeks prior from 0.52 to 0.58 ppm (Figure 2b4) corresponded to an estimated increase in ED admissions risk by 6% (IRR: 1.06, 95% CI: 1.10–1.12) to 17% (IRR: 1.17, 95% CI: 1.09–1.26). Significant positive associations were also estimated for increased CO concentrations 3 weeks prior, indicating that higher CO concentrations have both immediate and sustained effects on respiratory infection ED admissions.

### 3.4 Contribution of Exposures Toward Cause‐Specific ED Admissions

**Figure 3:**
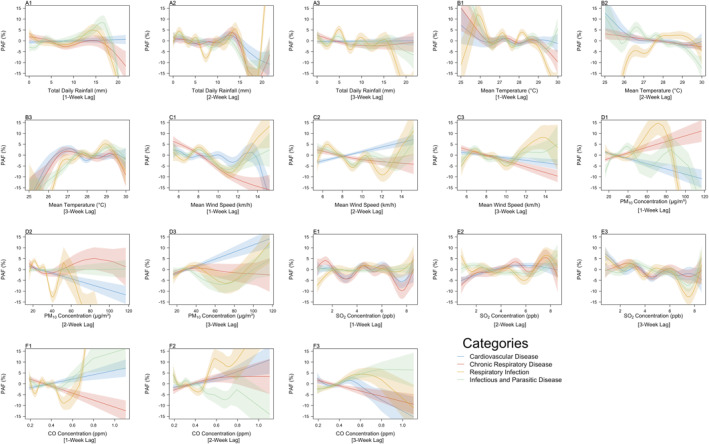
Population attributable fractions for (a1–a3) Total Daily Rainfall (mm), (b1–b3) Mean Temperature (°C), (c1–c3) Mean Wind Speed (km/h), (d1–d3) PM_10_ Concentration (μg/m^3^), (e1–e3) SO_2_ Concentration (ppb), (f1–f3) CO Concentration (ppm) at 1, 2, and 3‐week lags for the admission categories present in the Cardiorespiratory System Disorders group. The smoothed lines represent the trend observed from the population attributable fractions (PAFs), while the shaded regions represent the confidence intervals of the trends of the PAFs across the entire range of observed values for each respective exposure. For exact PAF values, refer to Figure S5 in Supporting Information S1.

Within the cardiorespiratory system disorders group, the trends of the PAFs for the respiratory infection and infectious and parasitic disease categories followed each other closely, while the PAFs of cardiovascular disease and chronic respiratory disease demonstrated slight deviation in comparison to the other categories present within the group. Total daily rainfall values above 15 mm lead to decreases in the predicted contribution to ED admissions, down to negative values (Figures 3a1–3a3), across the four categories and all three lags considered, indicating that rainfall above 15 mm provided protective effects against the diseases in these categories, with the exception of respiratory infections, where the exposure was predicted to contribute to almost 30% of the ED admissions (PAF: 29.1%, 95% CI: 22.2%–36.1%) as total daily rainfall exceeded 20 mm at a 2‐week lag (Figure 3a2). At both one and two week lags, increased mean temperature resulted in a general decrease in the predicted contributions across the categories (Figures 3b1–3b3), with the exception of respiratory infections, wherein the contribution of the exposure toward ED admissions increased drastically from −63.7.4% (95% CI: −74.7% to −52.6%) to 2.5% (95% CI 0.6%–4.4%), as the mean temperature increased from 25 to 29°C (Figure 3b2). This indicates that low temperatures (25–27°C) were predicted to contribute to fewer respiratory infection ED admissions. At a 3‐week lag, the predicted contributions for all four categories had an inverted U‐shaped curve (Figure 3b3), with the peaks for respiratory infections (PAF: 5.2%, 95% CI: 3.1%–5.2%) and infectious and parasitic diseases (PAF: 3.8%, 95% CI: 2.1%–5.4%) lying around 29°C, while that of cardiovascular diseases (PAF: 3.0%, 95% CI: 1.4%–4.6%) and chronic respiratory diseases (PAF: 1.8%, 95% CI: −0.1% to 3.79%) was between 26 and 27°C. The contribution of mean wind speed toward respiratory infection and infectious and parasitic diseases ED admissions were estimated to generally increase at high wind speeds (>12.5 km/hr) across all three lags considered (Figures 3c1–3c3). On the other hand, the contribution of the exposure toward cardiovascular disease and chronic respiratory disease ED admissions were estimated to be negative at high wind speeds (Figures 3c1–3c3), with the exception of contribution toward cardiovascular disease ED admissions at a 2‐week lag, where the predicted contribution was estimated to increase from −3.2% (95% CI: −4.3% to −2.2%) to 7.1% (95% CI: 4.8%–9.5%) as wind speeds increased from 5.43 to 15.24 km/hr (Figure 3c2). Ambient air pollutants displayed varying trends in the PAFs across the four categories, as well as the different lags considered. In general, PM_10_ concentrations around 70 μg/m^3^ were found to increase the predicted contribution toward respiratory infection ED admissions (PAF: 14.7%, 95% CI: 9.3%–20.1%) at a 1‐week lag (Figure 3d1), indicating an estimated immediate effect of PM_10_ concentrations on respiratory infection ED admissions. For the remainder of the categories however, increases in PM_10_ concentrations corresponded to increases in predicted contribution toward ED admissions only at a 3‐week lag (Figure 3d3), indicating a delayed effect for the remaining categories. SO_2_ concentrations ∼7 ppb were predicted to result in the increase in the predicted contributions toward chronic respiratory disease (PAF: 5.6%, 95% CI: 1.9%–9.3%) and respiratory infection (PAF: 6.6%, 95% CI: 2.9%–10.3%) ED admissions at a 2‐week lag (Figure 3e2), while predicted contributions toward ED admissions for the 1‐week (Figure 3e1) and 3‐week lags (Figure 3e3) across all categories were estimated to generally decrease with increases in SO_2_ concentrations. These indicate a slight delayed effect of increased SO_2_ concentrations on cardiovascular and respiratory system disorders. Increases in CO concentrations at a 1‐week lag were estimated to increase contributions toward respiratory infection, cardiovascular disease and infectious and parasitic diseases ED admissions (Figure 3f1). Meanwhile, increases in CO concentrations at a 2‐week lag were estimated to increase contributions toward respiratory infection, cardiovascular disease and chronic respiratory disease ED admissions (Figure 3f2). In particular, CO concentration was estimated to have a predicted contribution greater than 30% at CO concentrations above 0.9 mm at both lags (PAF: 50.1%, 95% CI: 40.0%–60.2% at 1‐week lag, PAF: 32.8%, 95% CI: 21.3%–44.3% at 2‐week lag) toward respiratory infection admissions, indicating a deleterious impact of CO concentrations on respiratory infections. At a 3‐week lag, however, the predicted contribution of CO concentration toward ED admissions across all four categories were found to generally decrease at CO concentrations >0.6 ppb (Figure 3f3). This indicates that the effect of increases in CO concentrations are estimated to be diminished beyond 2 weeks.

**Figure 4:**
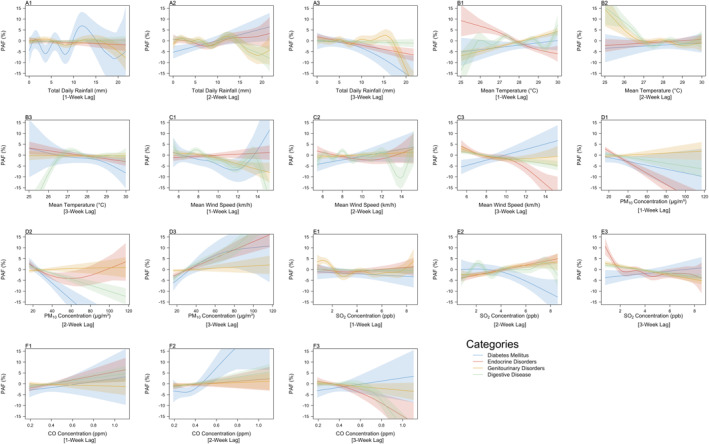
Population attributable fractions for (a1–a3) Total Daily Rainfall (mm), (b1–b3) Mean Temperature (°C), (c1–c3) Mean Wind Speed (km/h), (d1–d3) PM_10_ Concentration (μg/m^3^), (e1–e3) SO_2_ Concentration (ppb), (f1–f3) CO Concentration (ppm) at 1, 2, and 3‐week lags for the admission categories present in the Metabolic and Digestive System Disorders group. The smoothed lines represent the trend observed from the population attributable fractions (PAFs), while the shaded regions represent the confidence intervals of the trends of the PAF values of the exposure across the entire range of observed values for each respective exposure. For exact PAF values, refer to Figure S5 in Supporting Information S1.

Within the metabolic and digestive system disorders group, increases in total daily rainfall were predicted to contribute negatively toward ED admissions, with predicted contributions being estimated to generally decrease to negative values with increases in total daily rainfall (Figures 4a1–4a3), indicating a protective effect of high rainfall on the four categories present in this group. Diabetes mellitus and endocrine disorders displayed deviations from this general trend, as increases in total daily rainfall 2‐weeks prior lead to a predicted increase in contributions toward ED admissions for the two categories (Figure 4a2). Increased mean temperature (>28°C) 1‐week prior corresponded to increases in the predicted contribution up to 7.0% toward Genitourinary Disorders (PAF: 4.3%, 95% CI: 2.7%–5.9%) and Digestive Disease (PAF: 7.2%, 95% CI: 2.9%–12.1%) ED admissions (Figure 4b1), but a predicted negative contribution (PAF: −6.0%, 95% CI: −2.3% to −9.7%) toward Endocrine Disorders ED admissions. At 2 and 3‐week lags, the predicted contributions of temperature toward ED admissions were estimated to converge toward zero (Figures 4b2–4b3), indicating that temperature had a negligible effect on ED admissions for these categories at the given lags. Increased PM_10_ concentrations (>60 μg/m^3^) at a 3‐week lag were estimated to result in drastic increases in the predicted contribution of PM_10_ concentrations toward ED admissions, contributing toward 25.3%, 16.0%, and 9.3% of ED admissions when the PM_10_ concentration was 120 μg/m^3^ for digestive diseases (PAF: 25.3%, 95% CI: 18.8%–31.9%), endocrine disorders (PAF: 16.0%, 95% CI: 10.1%–21.8%) and diabetes mellitus (PAF: 9.3%, 95% CI: 0.3%–18.3%) categories (Figure 4d3). However, the predicted contribution of PM_10_ concentration toward the genitourinary disorders admission category remained stable at this value of PM_10_ concentrations, hovering around zero. These results indicate a delayed but significant impact of PM_10_ concentrations above 60 μg/m^3^ on the three categories. Similarly, high SO_2_ concentrations (>4 ppb) 2‐weeks prior corresponded to an increase in the predicted contribution of SO_2_ concentration toward ED admissions for the endocrine disorders category to 5% (95% CI: 2.7%–7.3%, Figure 4e2), while the predicted contribution toward the diabetes mellitus category was estimated to be negative (PAF: −12.7%, 95% CI: −4.5% to −20.9%), indicating a delayed and protective effect of increases in SO_2_ concentrations at this range. High CO concentrations (>0.6 ppm) 1 week prior were predicted to increase the contribution of CO concentration toward ED admissions of digestive disease (PAF: 4.7%, 95% CI: 1.1%–8.2%) and endocrine disorders (PAF: 3.6%, 95% CI: 0.3%–6.9%) categories (Figure 4f1), indicating a weak effect of the pollutant on these two categories, as the predicted contributions of the exposure were estimated to only reach a maximum of approximately 4%. However, at a 3‐week lag, high CO concentrations resulted in an estimated drastic decrease in predicted contributions of the exposure toward ED admissions of the same two categories (PAF: −26.3%, 95% CI: −18.5% to −34.1%) for digestive disease, PAF: −18.0%, 95% CI: −8.5% to −27.4% for endocrine disorders, to below −15% (Figure 4f3). These indicate that high CO concentrations have a protective effect on the two admission categories within this group at a 3‐week lag.

**Figure 5:**
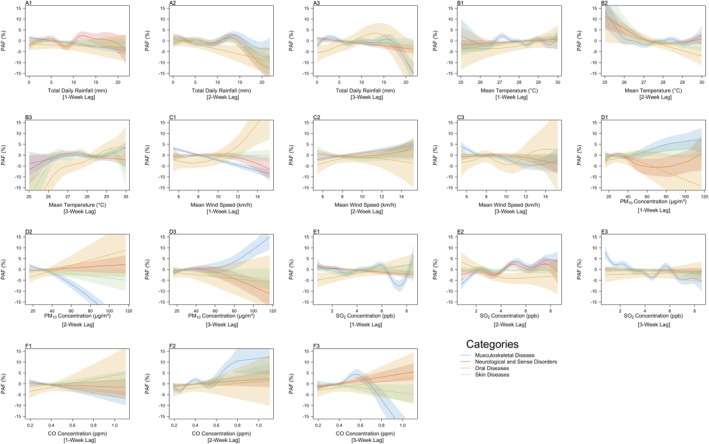
Population attributable fractions for (a1–a3) Total Daily Rainfall (mm), (b1–b3) Mean Temperature (°C), (c1–c3) Mean Wind Speed (km/h), (d1–d3) PM_10_ Concentration (μg/m^3^), (e1–e3) SO_2_ Concentration (ppb), (f1–f3) CO Concentration (ppm) at 1, 2, and 3‐week lags for the admission categories present in the “Others” group. The smoothed lines represent the trend observed from the population attributable fractions (PAFs), while the shaded regions represent the confidence intervals of the trends of the PAF values of the across the entire range of observed values for each respective exposure. For exact PAF values, refer to Figure S5 in Supporting Information S1.

For the remaining ED admissions due to other disorders, high rainfall values (>15 mm) were estimated to correspond to negative contributions toward ED admissions for all the categories (Figures 5a1–5a3), across all three lags considered, indicating that higher rainfall provided a protective effect against ED admissions for these categories. Temperatures below 28°C one week and three weeks prior corresponded to estimated negative contributions toward ED admissions within the group (Figures 5b1 and 5b3), similarly indicating a protective effect of temperature at the given range, with the exception of the Musculoskeletal Disease category (PAF: 2.3%, 95% CI: 0.9%–3.6% at a temperature of 27.1°C 1‐week prior). However, at a two week lag temperatures below 27°C corresponded to estimated positive contribution toward ED admissions for all categories, with the predicted contributions reaching values above 10% for the Skin Diseases (PAF: 17.8%, 95% CI: 9.3%–26.3%) and Neurological and Sense Disorders (PAF: 11.5%, 95% CI: 5.9%–17.1%) categories (Figure 5b2), indicating a delayed effect of temperature on these particular admission categories. Wind speeds greater than 10 km/hr 1 week prior corresponded to an estimated increase in the predicted contribution to beyond 10% for ED admissions in the Oral Diseases category (PAF: 11.7%, 95% CI: 4.6%–23.6%, Figure 5c1), which indicates a significant contribution toward ED admissions. While the estimated contributions of wind speed toward ED admissions at two and 3 week lags had both protective and deleterious effects across all four admissions categories (Figures 5c2–5c3), the predicted contributions of the exposure were generally low, ranging between −2% and 2%, indicating poor contributions toward the admissions for the respective categories. Increases in PM_10_ concentrations resulted in an estimated increase in predicted contributions toward ED admissions for the musculoskeletal disease category (PAF: 7.0%, 95% CI: 1.2%–12.8% at a 1‐week lag and PAF: 10.4%, 95% CI: 6.5%–14.3% at a 3‐week lag) beyond 5% (Figures 5d1 and 5d3), while the predicted contribution of PM_10_ concentrations for the remaining admission categories remained close to 0%. Similarly, high SO_2_ concentrations (>4 ppb) only corresponded to an increase in predicted contribution toward ED admissions for the musculoskeletal disease category (PAF: 4.5%, 95% CI: 2.0%–6.9%, Figure 5e2), while the predicted contributions of high SO_2_ concentrations for the other categories and at the other lags did not deviate much from 0%. Additionally, high CO concentrations (>0.9 ppm) 2 weeks prior (Figure 5f2) corresponded to a predicted contribution of up to 9% of ED admissions for the musculoskeletal disease category (PAF: 9.0%, 95% CI: 5.6%–12.5%), while the predicted contribution toward ED admissions for the remaining categories were estimated to between −2% and 2% (Figures 5f1–5f3) at the given range of CO concentrations, indicating that ambient air pollutants in general disproportionately affect the musculoskeletal disease category in comparison to the other categories present in this group.

## 4 Discussion

Analyses of the non‐linear associations and contributions between meteorological factors, ambient air pollutants with 12 ED admissions categories over the 5‐year study period demonstrated that the lagged exposures considered in this study had significant impacts on, and consistently influenced cause‐specific ED admissions. Although it was found that certain factors affected the categories disproportionately, our analyses revealed several findings that can be generally applied: (a) high total daily rainfall was negatively associated with ED admissions (b) mean temperature, while positively associated with ED admissions, typically had delayed effects on ED admissions (c) increased wind speed contributed disproportionately to increased ED admissions due to respiratory infections (d) increased ambient air pollutant concentrations while positively associated with ED admissions due to most categories, had the greatest impact on the categories belonging to cardiorespiratory system disorders.

### 4.1 Effect of Total Daily Rainfall

Rainfall is a factor that has been extensively studied, in particular for the resulting heightened risk of the transmission of several infectious diseases. The variations in precipitation patterns are known to impact their spread, as well as likelihood of evolving into epidemics (Chen et al., 2012; Greenough et al., 2001; McMichael et al., 2006). Moreover, in times of heavy rainfall, the quality of local water can be significantly compromised through various mediums, and has been linked to ED admissions for acute gastrointestinal illness (Drayna et al., 2010). Most of the categories considered in our study did not display positive associations with rainfall, which could be a consequence of under reporting as people are less likely to visit hospitals during bouts of heavy rain. However, the findings presented in works above do corroborate with our analyses of the respiratory infections and infectious and parasitic disease categories. Our findings indicate positive associations with increased rainfall and infectious diseases, which can be attributed to several reasons. For example, vector‐borne infectious diseases such as dengue are known to be affected by the patterns of rainfall, as heavy rainfall results in pools of stagnant water, providing ideal breeding conditions for the *Aedes Aegypti* vector (do Nascimento et al., 2022). Our findings are concordant with previous work (Lim et al., 2020; H. Sun et al., 2021; Tewari et al., 2023) that has investigated the epidemiological effects of rainfall on dengue transmission. Rainfall also affects human behavior, causing people to stay indoors or gather in shelters, thus promoting the transmission of infectious diseases between individuals. The discrepancy of the protective effects of rainfall indicated by our study for the remaining categories, and the work of Drayna et al. (2010), which found associations between gastrointestinal illness and heavy rainfall can be explained by the quantity of rainfall considered. In their study, the positive association was found during extreme rainfall events, which was defined as rainfall exceeding 2.54 cm in a 24‐hr period, which is considerably higher than the rainfall observed in our study. The lack of such heavy rainfall locally, as well as the presence of good water management practices thus diminishes the chances of sewage overflow in our study setting, which Drayna et al. had attributed to the increases in ED admissions. Thus, under non‐extreme weather events, rainfall has a negative association with ED admissions across most of the admission categories considered in our study.

### 4.2 Effect of Temperature

Exposure to high ambient temperature and extreme levels of heat has been recognized as a public health hazard (Weinberger et al., 2020), with the adverse impacts of heat on renal (Kingsley et al., 2016), cardiorespiratory (Anderson et al., 2013; Michelozzi et al., 2009) and mental (Liu et al., 2021) illness being well documented in scientific literature. Similarly, our study estimated positive associations between higher ambient temperatures and the cardiovascular disease, digestive disease and genitourinary disorders (Refer to Figure S4 in Supporting Information S1) ED admission categories. Our analyses are consistent with the robust body of evidence that exposure to heat increases morbidity. For example, an analysis of US Medicare beneficiaries aged 65 years and older between the study period of 1992–2006 reported that instances of extreme heat, which was defined as a day with apparent temperature at the 99th city‐specific temperature centile versus the 75th centile, correlated with a 3.2% (95% CI: 2.4%–4.0%) increased likelihood of ED admission for any cause (Gronlund et al., 2014). High levels of heat are potent as they can outpace a person's ability to thermoregulate, placing stresses on the metabolic system. Yet, the estimated contribution of temperature toward ED admissions for categories found within the metabolic system disorders group (Figure 4) was not estimated to be significantly higher than the other categories considered in this study, which hint at the potential for heat to place stresses across multiple physiological systems. While our results have been consistent with the existing literature in certain aspects, there is a disparity in our findings of the delayed effect of heat on ED admissions. For example, studies carried out in Brazil (Zhao et al., 2019) and the work of Gronlund et al. (2014) studied the positive associations between heat and morbidities by considering only up to 8 days of lag, finding immediate effects of heat on morbidity. Although Gronlund's work failed to observe a decline in the effects of extreme heat in the 7 days following the day of extreme heat, it remains difficult to directly compare estimates across studies given the use of different exposure metrics, time periods, study population and natural local climate conditions. It is possible that the delayed onset of morbidities caused by higher temperatures are a result of the persistent warm local climate experienced in our study setting.

### 4.3 Effect of Windspeed

In our study, associations between windspeed and ED admissions displayed varying directions within the different admission categories considered, as well as the different time lags considered. The only discernible and consistent pattern that emerged from considering both IRRs (Refer to Figure S4 in Supporting Information S1) and PAFs (Figures 3c1–3c3) of windspeed was the finding that increased windspeeds were consistently positively associated with increased ED admissions due to respiratory infections and oral diseases. Our analysis revealed a disproportionate impact of increased wind speed on the surge of respiratory infection‐related ED admissions, with high wind speed being attributable for 10% of the cases at a 1‐week lag (Figure 3c1). Studies conducted during the COVID‐19 pandemic have consistently reported positive associations between increased wind speeds and the transmission of COVID‐19 (Aidoo et al., 2021; Ali et al., 2021; Endeshaw et al., 2022; Şahin, 2020) and other respiratory infections (Li et al., 2022; Wenfang et al., 2020). Specifically, Endeshaw et al. (2022) reported that a 1 m/s increase in wind speed was associated with a 1.8‐fold increase in COVID‐19 confirmed cases. These findings further validate the positive associations found in our study between high wind speeds and respiratory infection. The positive associations found between respiratory infections and windspeed can be potentially explained by the increase in circulation of suspended respiratory droplets in the air due in the presence of strong winds, which typically serve as the medium for transmission of the infections (Y. Sun et al., 2009; H. Sun et al., 2021; Tewari et al., 2023; Tian et al., 2018). Similarly, the positive associations between increased wind speeds and oral diseases could be explained by the increased circulation of droplets containing enteroviruses that are responsible for oral conditions such as hand, foot and mouth disease (HFMD). A case study in China found a significant positive correlation between wind speed and HFMD incidence for 2 weeks prior in the counties within a 50 km buffer circle of the study setting (Liao et al., 2015). Wind speed may also influence the diffusion rates and distance spread of viruses, leading to increased proliferation of the infections or diseases.

### 4.4 Effect of Air Pollutants

The findings of our study underscore the deleterious impacts of ambient air pollutants on human health. High levels of air pollutant concentrations were found to be generally positively associated with ED admissions, placing particular stress on the cardiorespiratory system. Particulate Matter, a significant constituent of air pollution in urban environments, has links to various cardiorespiratory ailments, notably acute lower respiratory tract infections (Horne et al., 2018). Particulate Matter has adverse effects on human health, as it is known to potentially damage bronchial immunity and epithelial cell integrity (Jiang et al., 2020), which consequentially reduces the effectiveness of the body's immune response, making affected individuals more vulnerable to respiratory. These are consistent with the findings in our study, where categories in the cardiorespiratory system disorders group were found to be the most sensitive to increases in PM_10_ concentrations. The positive associations between the air pollutants and the cause‐specific admissions align with the established epidemiological and biological relationships extensively documented in various studies (Ghada et al., 2021; Goodman et al., 2004; Ho et al., 2022; Mills et al., 2009). For example, a study conducted across 218 Chinese cities reported positive associations between daily hospital admissions and several air pollutants (Tian et al., 2018). Tian et al. (2018) found that each 10 μg/m^3^ increase in SO_2_, and NO_2_, and 1 mg/m^3^ increase in CO at lag 0 day was associated with a 1.16% (95% CI: 0.92%–1.40%), 1.68% (95% CI: 1.40%–1.95%), and 2.59% (95% CI: 1.69%–3.50%) higher daily hospital admissions, respectively, which are similar to the findings in our work. While the links between air pollution and cardiorespiratory diseases have a robust body of evidence to support them, the associations between pollutants and other admission categories still remain unclear. In our work, the high ambient air pollutant concentrations were found to consistently be highly attributable to ED admissions for the musculoskeletal disease category (Figures 5d–5f). The mechanism by which pollutants may affect musculoskeletal disorders, whether via direct effects on the musculoskeletal system or by wearing down of the immunity of the human body remains unknown. Further experimental studies are required to explore the physiological regulation mechanisms involved. It is also worth noting that several categories in the metabolic and digestive system disorders group, such as the diabetes mellitus category, appeared to be insensitive to changes in select air pollutant concentrations, and instances such as this occur for throughout the different lags considered. This could be potentially explained by the relatively good level of air quality present throughout the study period, preventing the manifestation of the adverse effects of higher air pollutant concentrations.

### 4.5 Limitations

Although our research has provided valuable insights, it is crucial to recognize and tackle multiple limitations that warrant consideration when interpreting the findings. First, as only ED admissions to public hospitals were recorded, there is an under‐reporting of ED case counts across all the admission categories as admissions to private hospitals have not been accounted for. This may result in the incomplete capture of the interactive effects of the exposures on the categories. While our study incorporated relevant exposures that were selected after a comprehensive literature review, our modeling process is still subject to omitted variable bias, which could potentially lead to our models incorrectly attributing the effects of the omitted variables to the exposures chosen in this study. Our study was also carried out in a tropical climate setting over a 5‐year period, which may be insufficient to capture both year‐to‐year associations and the extreme weather variability that other geographical regions may experience. 2015 and 2016 were also years where major transboundary haze occurred, which significantly affected air quality in Singapore, which could potentially affect the interpretation of impacts of air pollutants. As such, our findings may not be employed to other study settings as well. Finally, while the associations presented in our study are consistent with other bodies of research, they do not imply causality. The association reported could be due to unmeasured confounders, which are difficult to account for.

## 5 Conclusion

Associations between meteorological factors, ambient air pollutants and ED admissions vary across the different categories and lags considered. However, the increasing effects of climate change on meteorological factors such as increasing ambient temperatures, inconsistent rainfall patterns and the increasing levels of ambient air pollution are poised to increase the strain on emergency departments. This indicates the pertinent need for more accurate and efficient resource planning to meet the impending challenges of healthcare systems worldwide. While the cardiorespiratory systems are heavily affected by these environmental factors, attention should also be paid to uncover the lesser understood impact of the exposures on the physiological mechanisms of the remaining admission categories.

## Conflict of Interest

The authors declare no conflicts of interest relevant to this study.

## Supporting information

Supporting Information S1

## Data Availability

All code required to reproduce this study is available at https://github.com/prnvtwr/ED‐Admissions (Tewari, 2024), accessed on 1 March 2024, and is deposited at https://doi.org/10.5281/zenodo.11517258.
