# Supplementary material for: Associations Between Anthropogenic Factors, Meteorological Factors, and Cause‐Specific Emergency Department Admissions
Source: Geohealth. 2024 Sep 4;8(9):e2024GH001061. doi: 10.1029/2024GH001061 (PMC11375029; doi:10.1029/2024GH001061)
Supplement: Supplementary file 1 — Supporting Information S1 [file GH2-8-e2024GH001061-s001.pdf]

AGU PUBLICATIONS

GeoHealth

Supporting Information for

Associations between anthropogenic factors, meteorological factors and cause-specific emergency department admissions

Pranav Tewari[1], Xu Baihui[1], Ma Pei[2], Kelvin Bryan Tan[3], John Abisheganaden[4], Steve Hung-Lam Yim[5], Borame Lee Dickens[2], Jue Tao Lim[1]

[1]Lee Kong Chian School of Medicine, Nanyang Technological University, Singapore, [2]Saw Swee Hock School of Public Health, National University of Singapore, Singapore, [3]Ministry of Health, Singapore, [4]Tan Tock Seng Hospital, Singapore, [5]Asian School of the Environment, Nanyang Technological University, Singapore

Contents of this file

Introduction
Figures S1 to S5
Table S1

Introduction

This supporting information provides figures/tables that are referenced in the main manuscript.

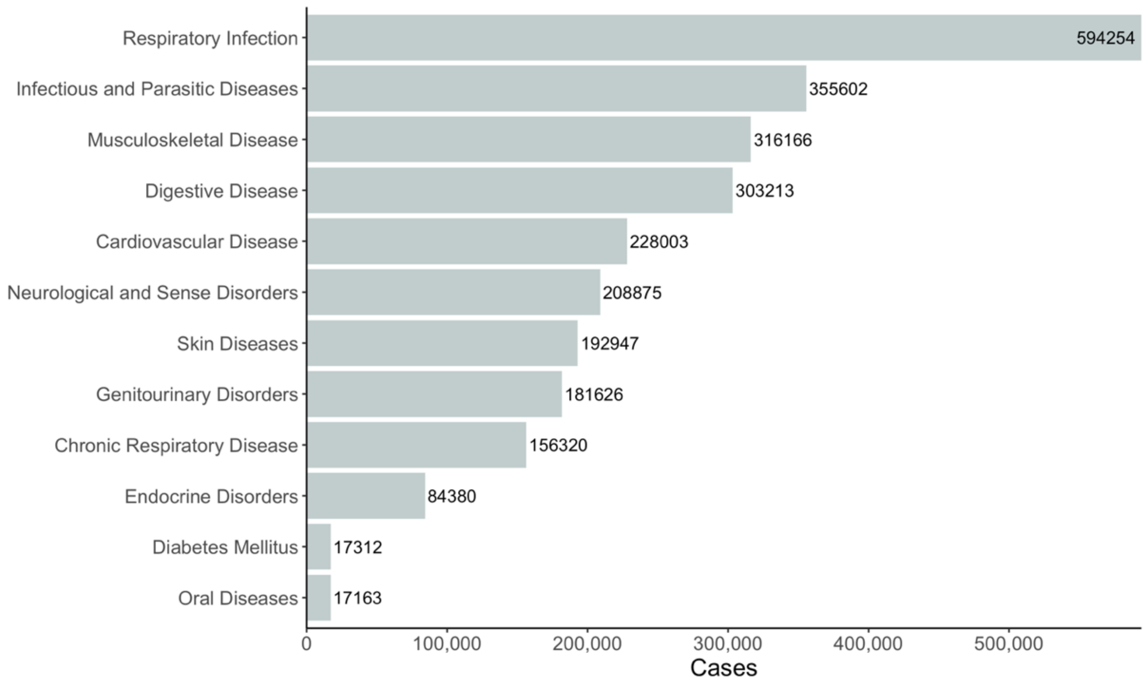

Figure S1. Total number of ED admissions across the study period of EW1 2014 to EW 52 2018 for each admission category in the study

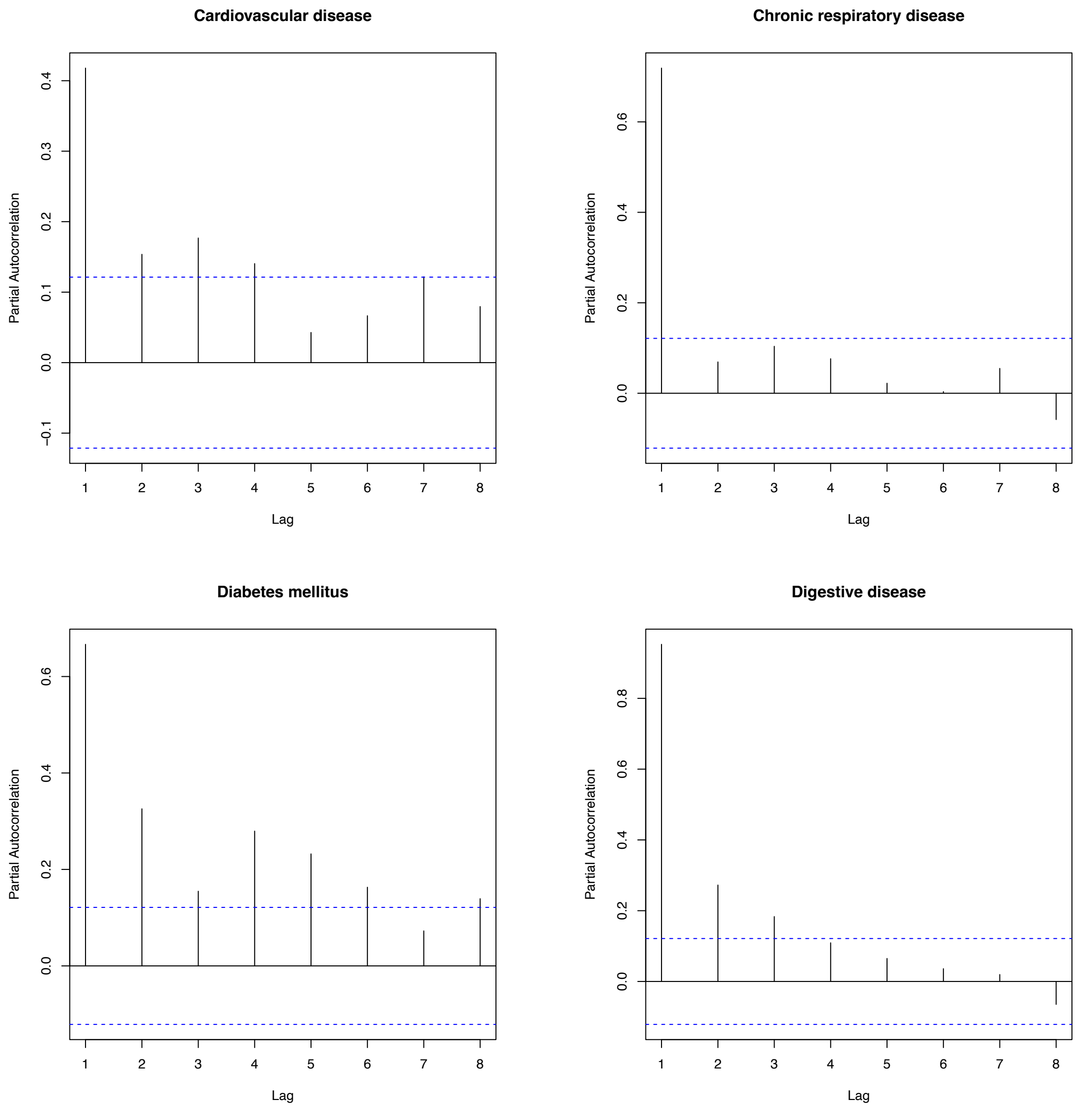
Cardiovascular disease
Chronic respiratory disease
Diabetes mellitus
Digestive disease
Partial Autocorrelation
Lag
1
2
3
4
5
6
7
8
0.4
0.3
0.2
0.1
0.0
−0.1
0.6
0.8

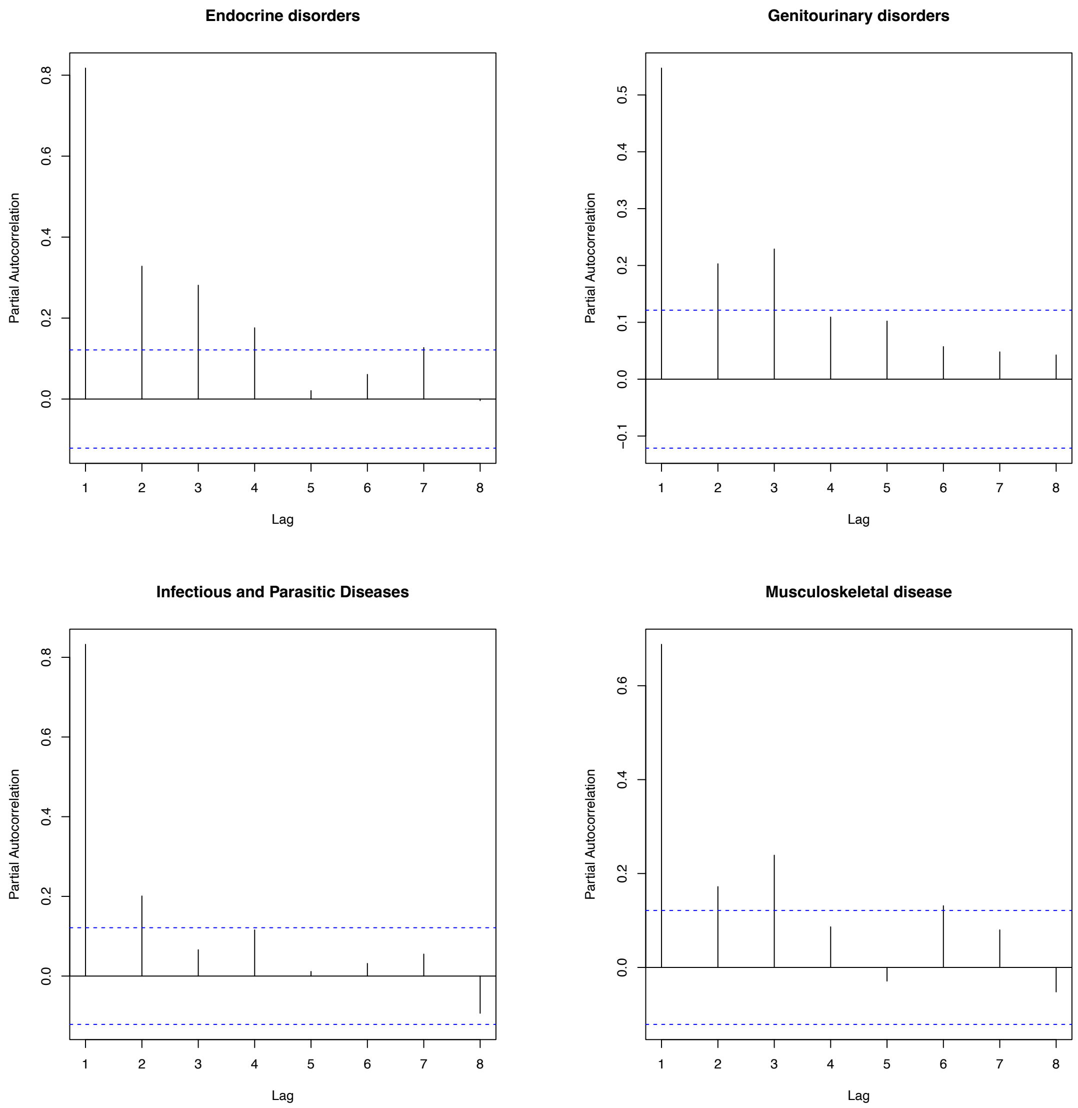
Endocrine disorders
Genitourinary disorders
Infectious and Parasitic Diseases
Musculoskeletal disease
Partial Autocorrelation
Lag
0.0
0.2
0.4
0.6
0.8
−0.1
0.1
0.3
0.5
1
2
3
4
5
6
7
8

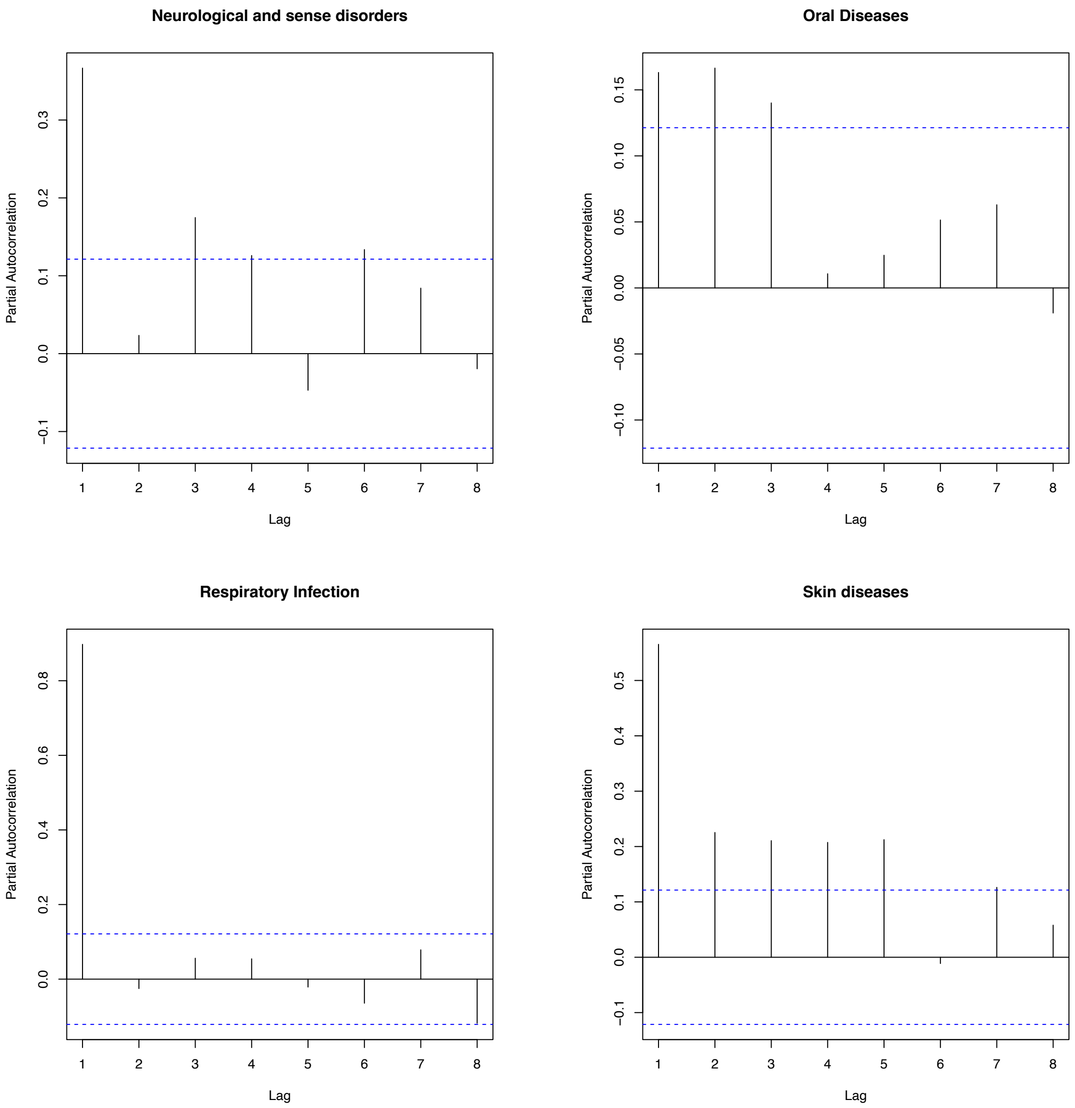

Figure S2. Partial autocorrelation plots over lags of up to 8 weeks for each admission category. Dashed blue lines represent 5% significance limits for the partial autocorrelations.

Chronic Respiratory Disease

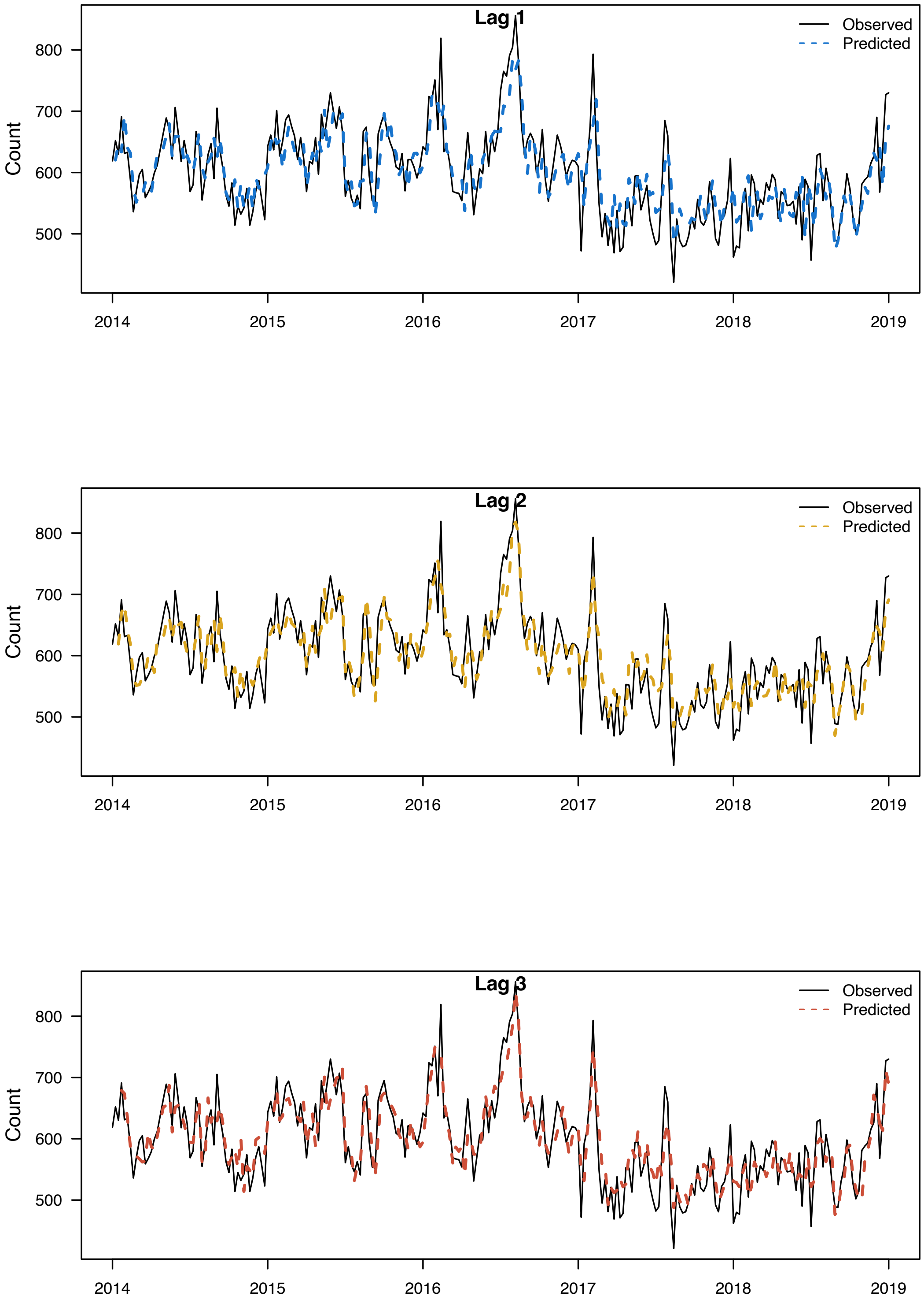

Diabetes Mellitus

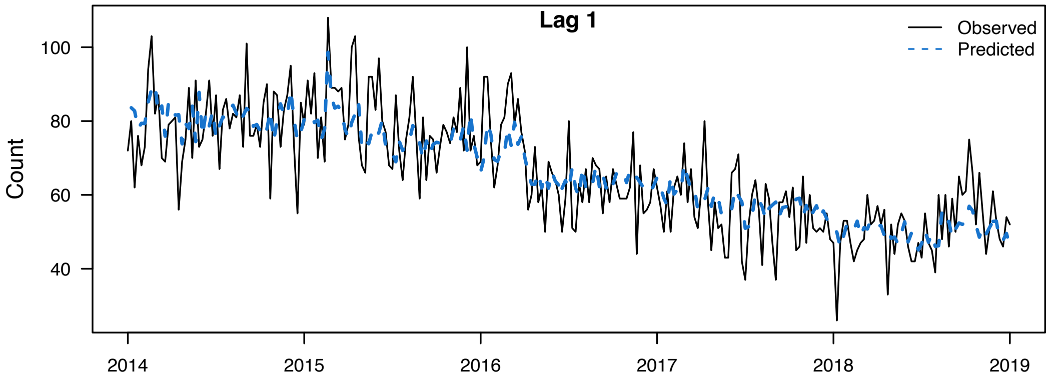

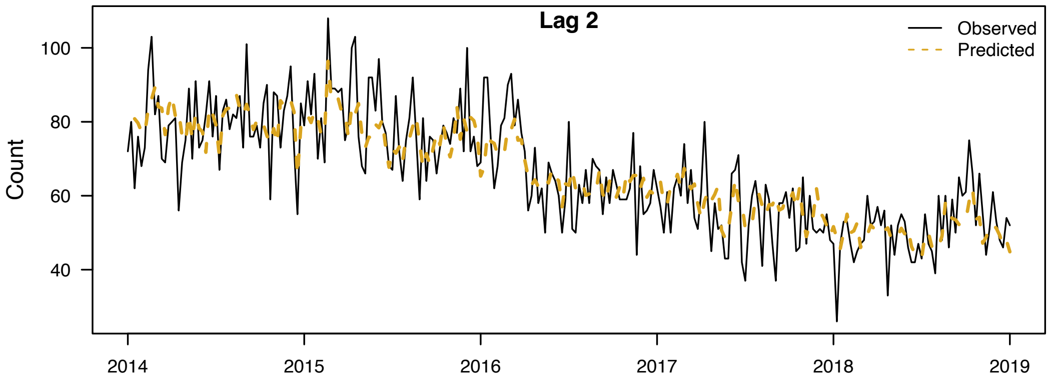

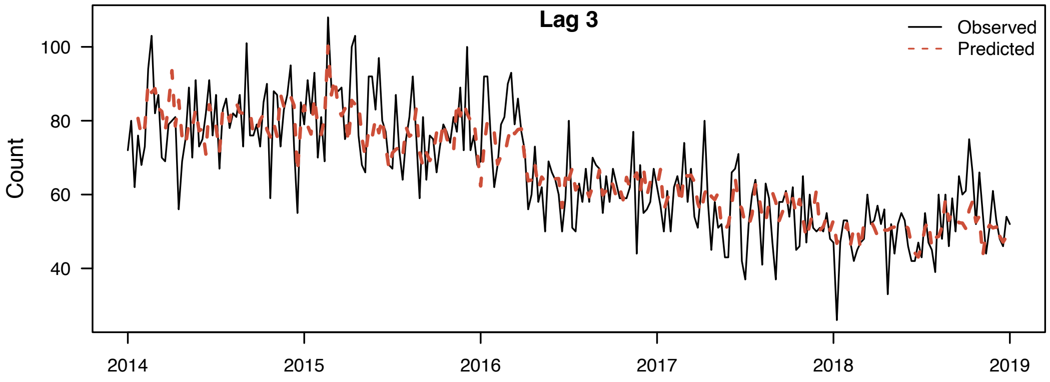

Digestive Disease

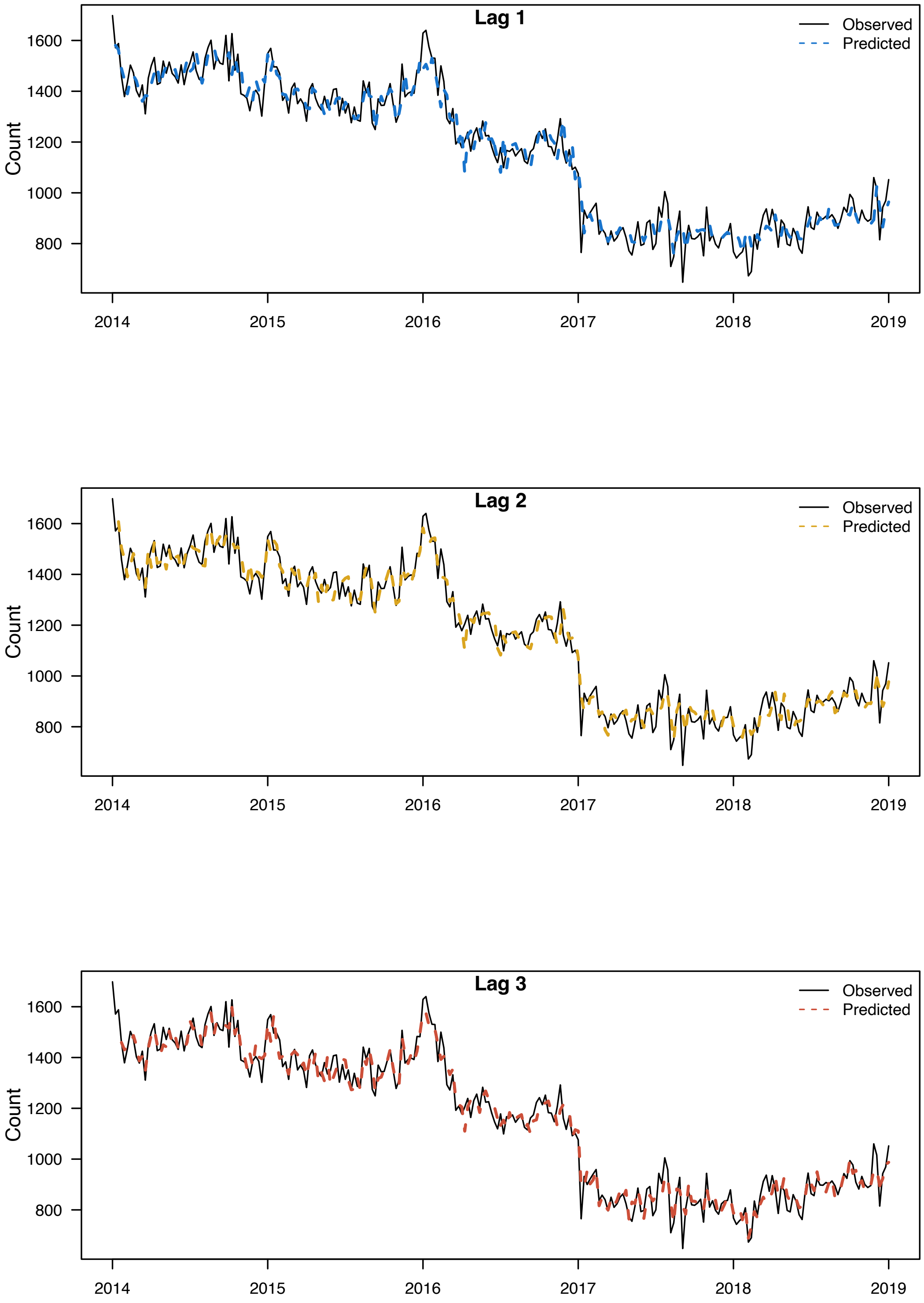

Endocrine Disorders

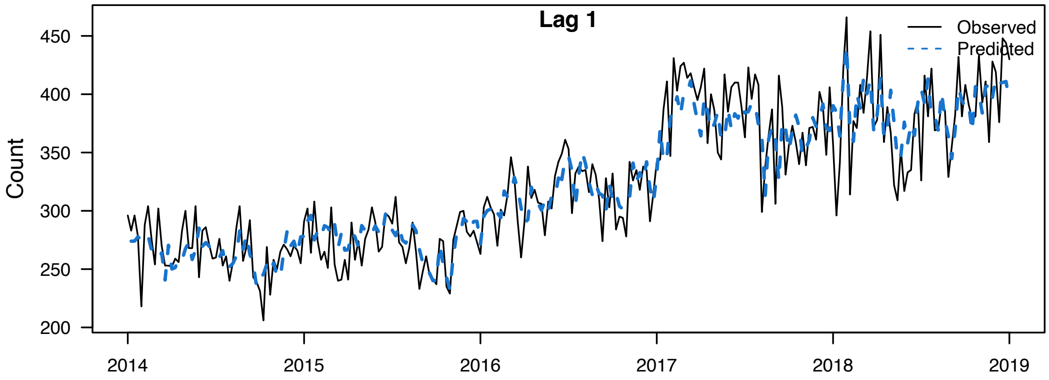

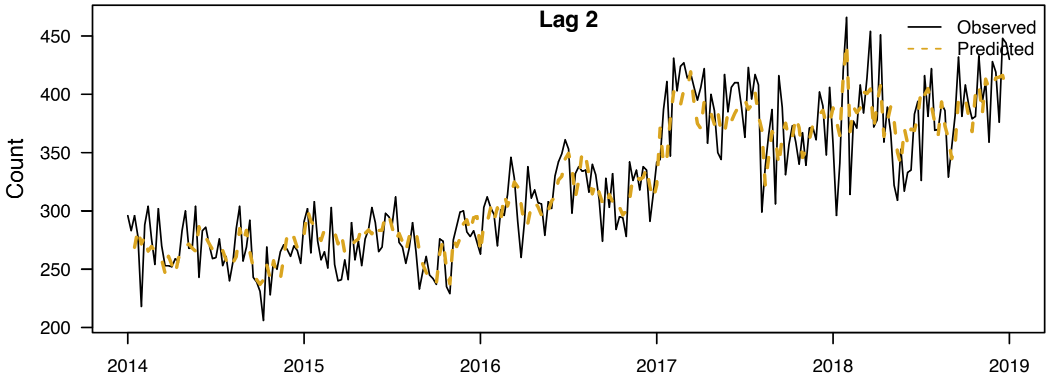

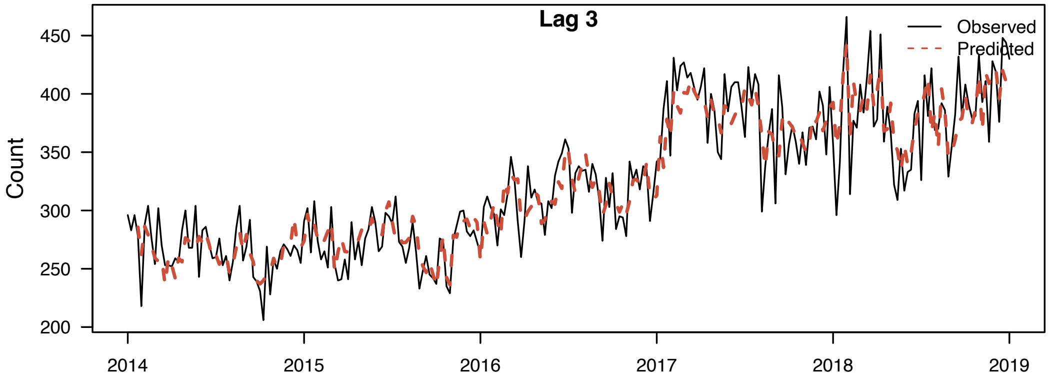

Genitourinary Disorders

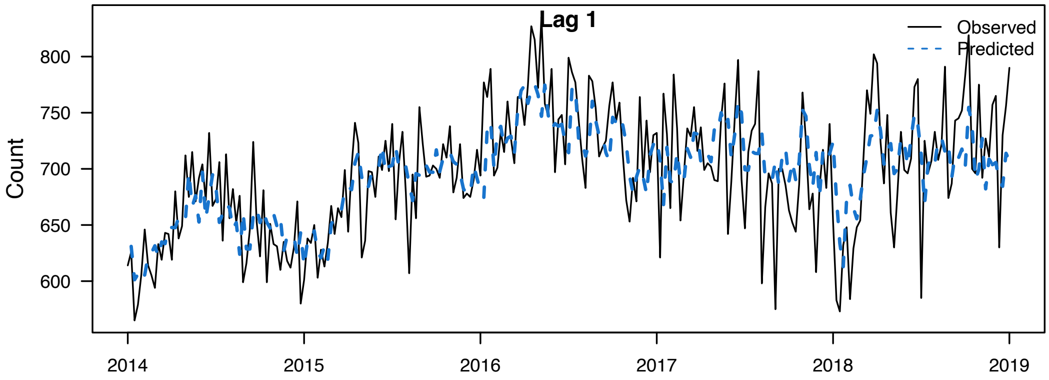

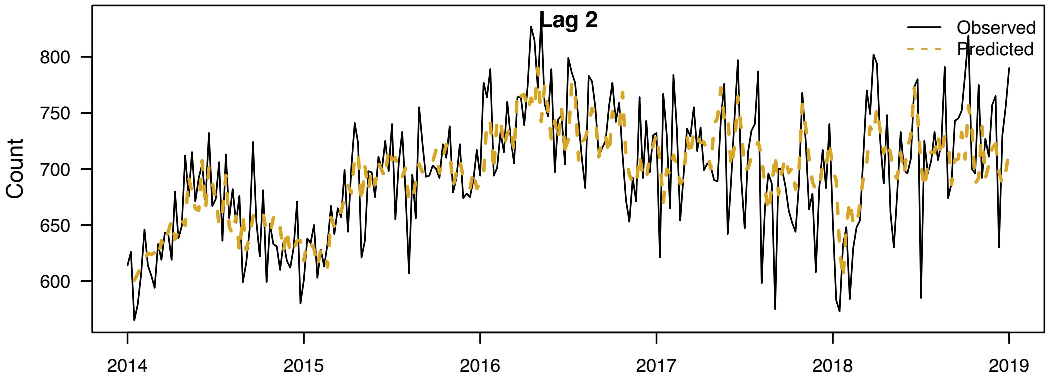

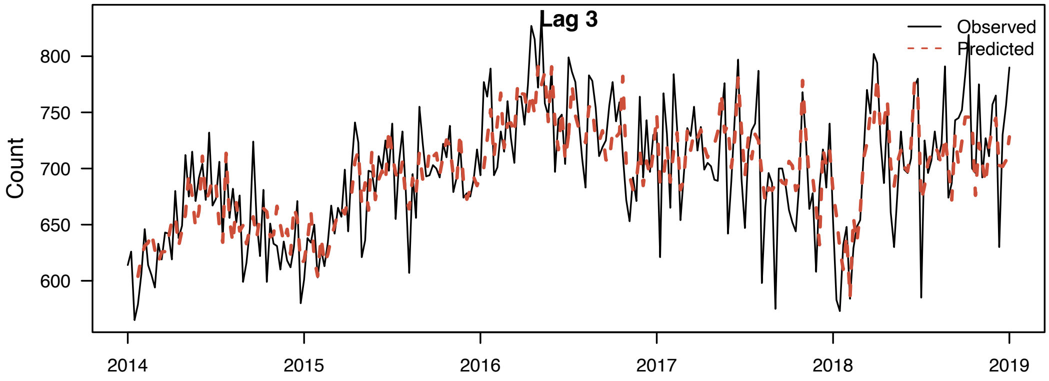

Infectious and Parasitic Diseases

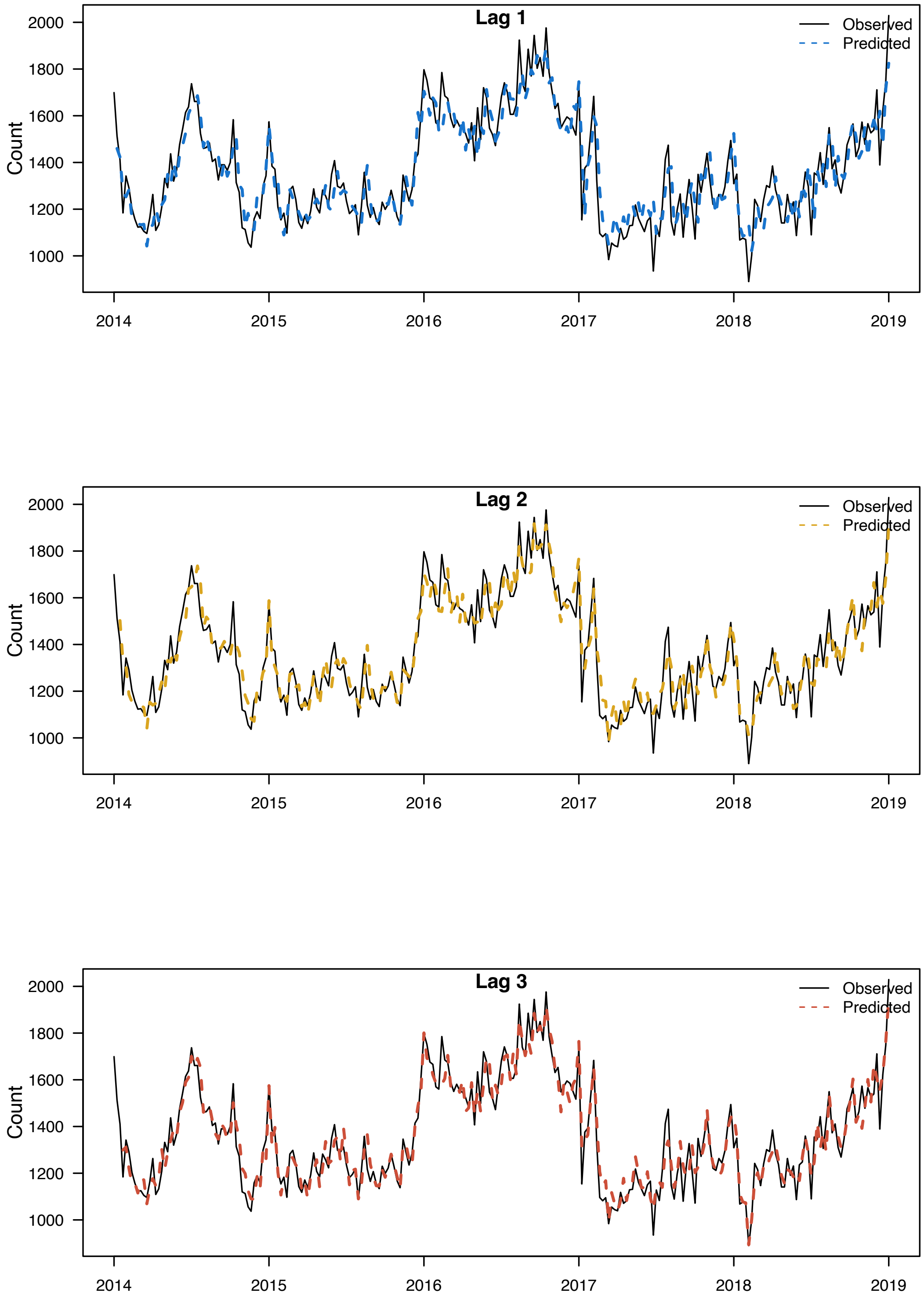

Musculoskeletal Disease

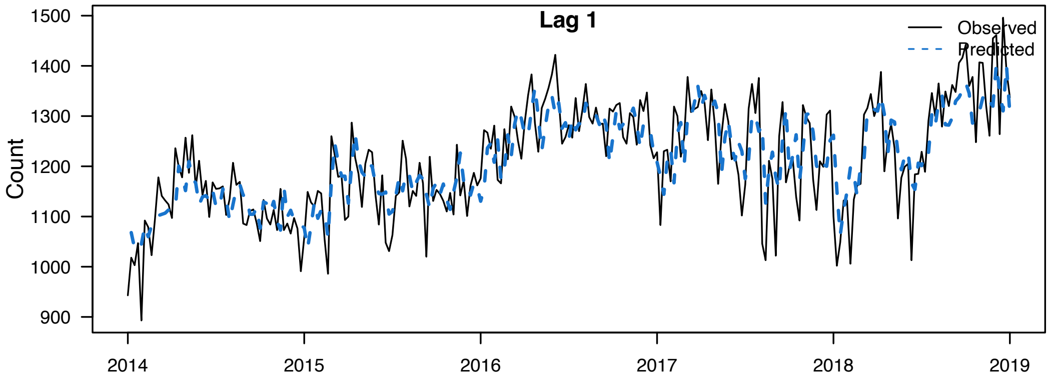

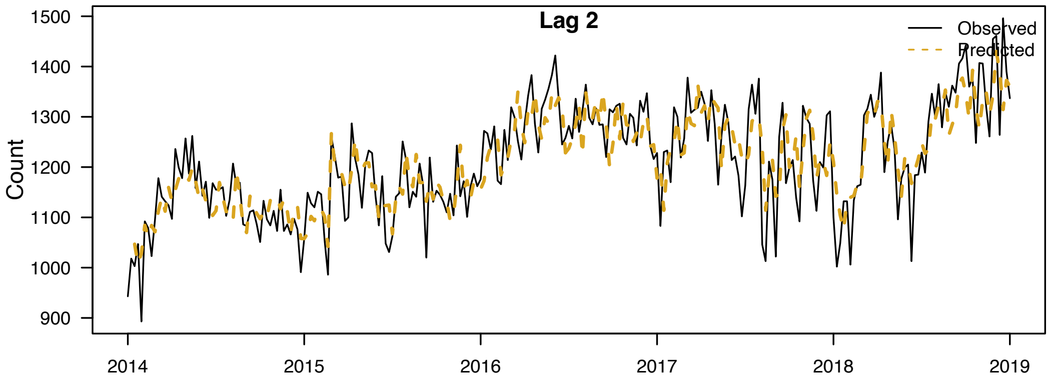

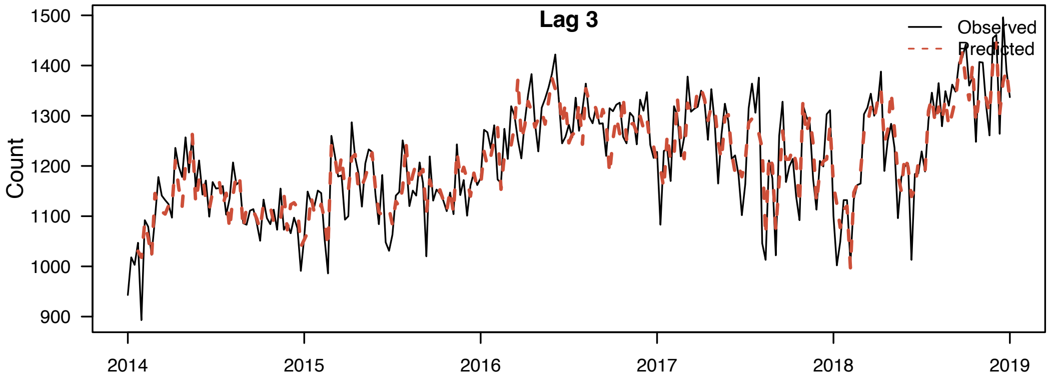

Neurological and Sense Disorders

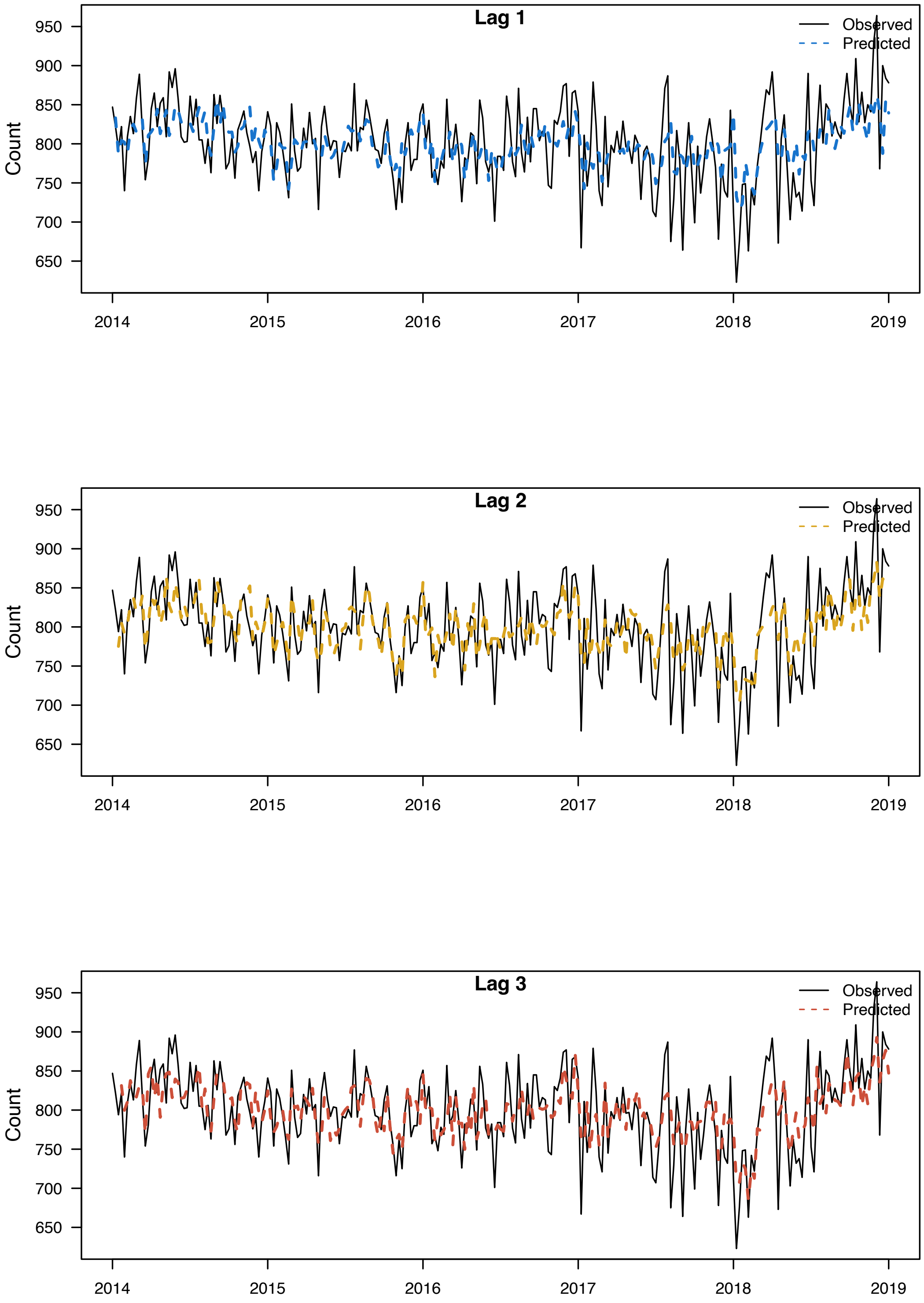

Oral Diseases

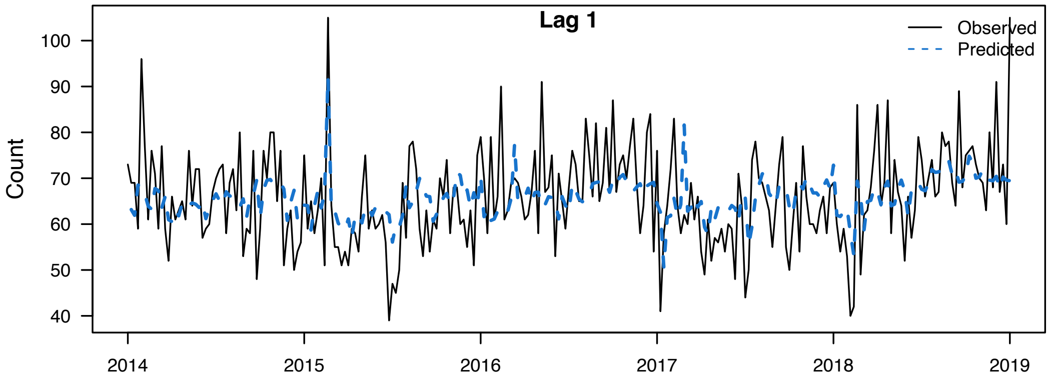

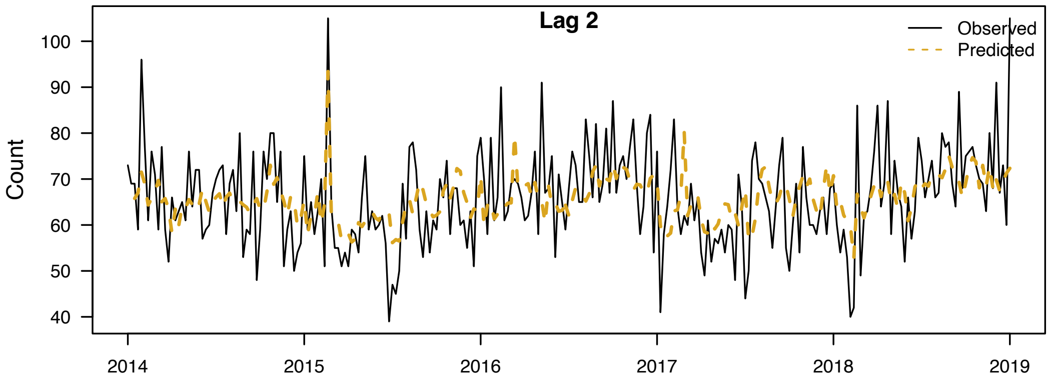

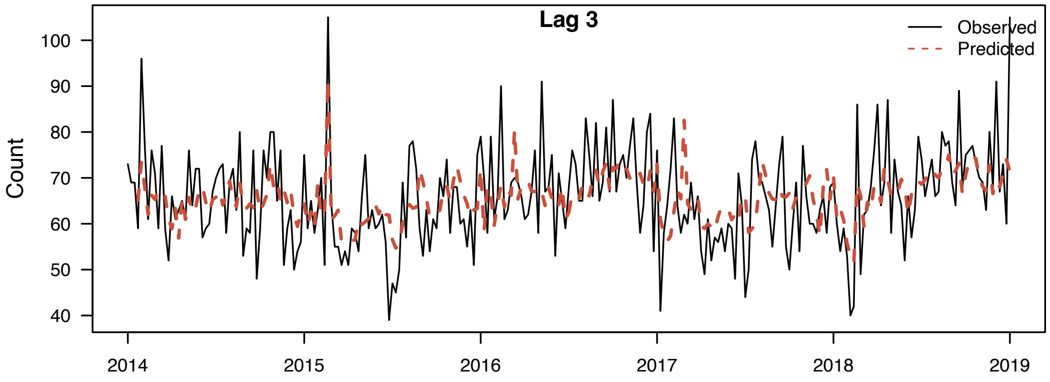

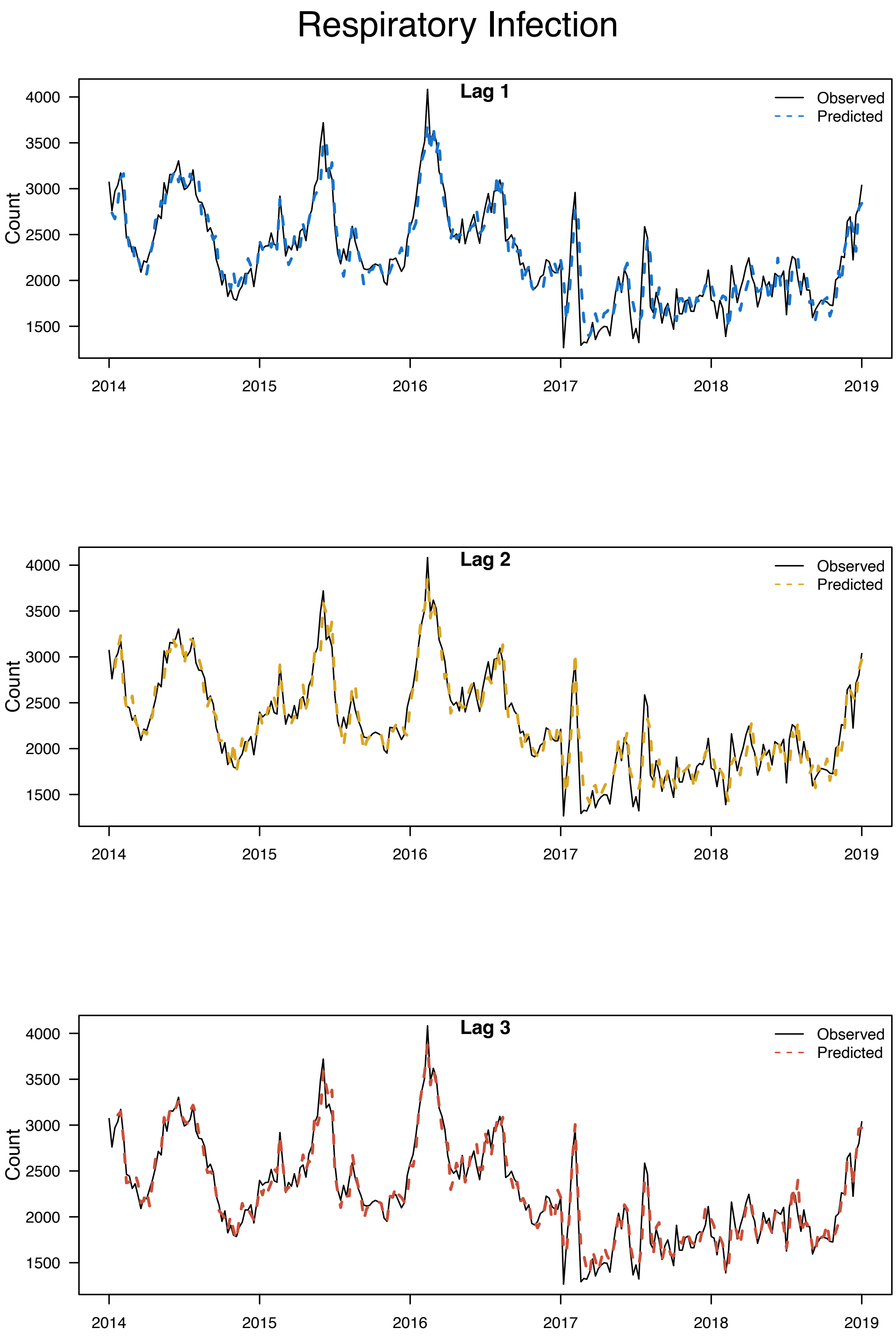

Respiratory Infection
Lag 1
Observed
Predicted
Count
4000
3500
3000
2500
2000
1500
2014
2015
2016
2017
2018
2019
Lag 2
Observed
Predicted
Count
Lag 3
Observed
Predicted
Count

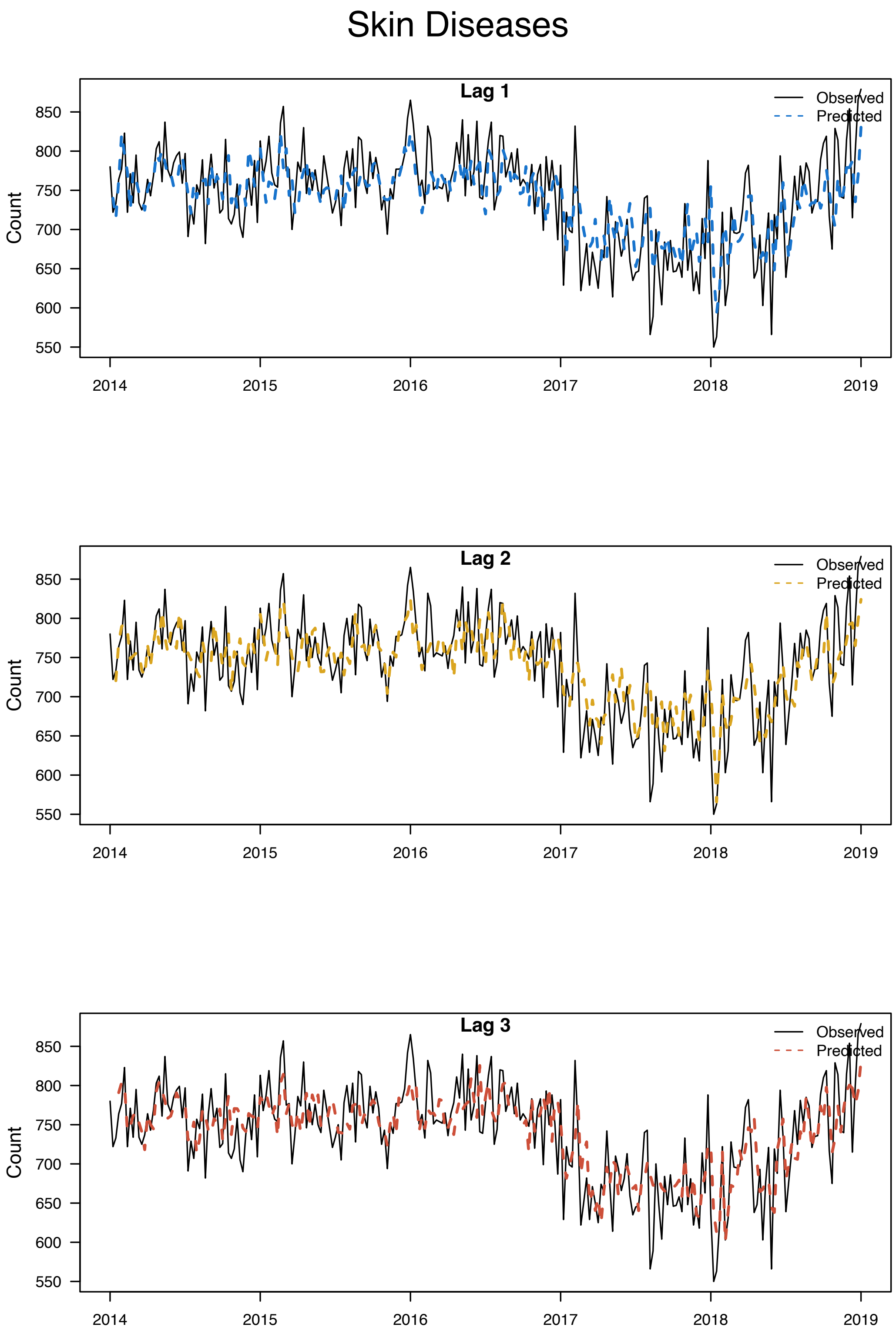

Figure S3. Observed (solid,black) and predicted (dashed, coloured) counts of ED admissions across each admission category. Predicted counts are plotted for each of the models with differing autoregressive terms considered.

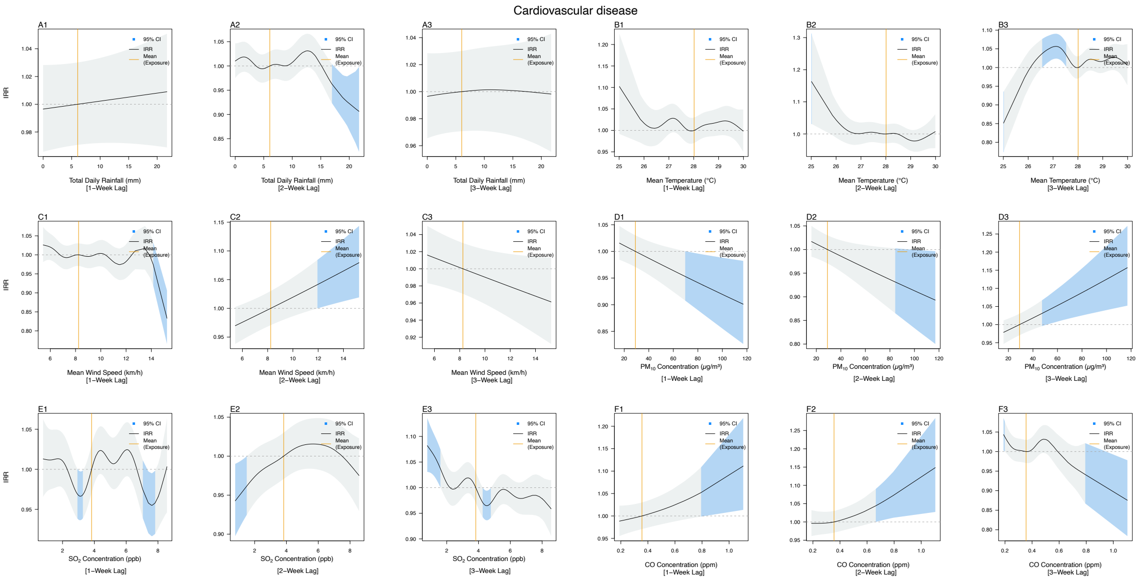
Cardiovascular disease
A1
A2
A3
B1
B2
B3
C1
C2
C3
D1
D2
D3
E1
E2
E3
F1
F2
F3
95% CI
IRR
Mean (Exposure)
IRR
Total Daily Rainfall (mm) [1–Week Lag]
Total Daily Rainfall (mm) [2–Week Lag]
Total Daily Rainfall (mm) [3–Week Lag]
Mean Temperature (°C) [1–Week Lag]
Mean Temperature (°C) [2–Week Lag]
Mean Temperature (°C) [3–Week Lag]
Mean Wind Speed (km/h) [1–Week Lag]
Mean Wind Speed (km/h) [2–Week Lag]
Mean Wind Speed (km/h) [3–Week Lag]
PM_{10} Concentration (μg/m³) [1–Week Lag]
PM_{10} Concentration (μg/m³) [2–Week Lag]
PM_{10} Concentration (μg/m³) [3–Week Lag]
SO_2 Concentration (ppb) [1–Week Lag]
SO_2 Concentration (ppb) [2–Week Lag]
SO_2 Concentration (ppb) [3–Week Lag]
CO Concentration (ppm) [1–Week Lag]
CO Concentration (ppm) [2–Week Lag]
CO Concentration (ppm) [3–Week Lag]

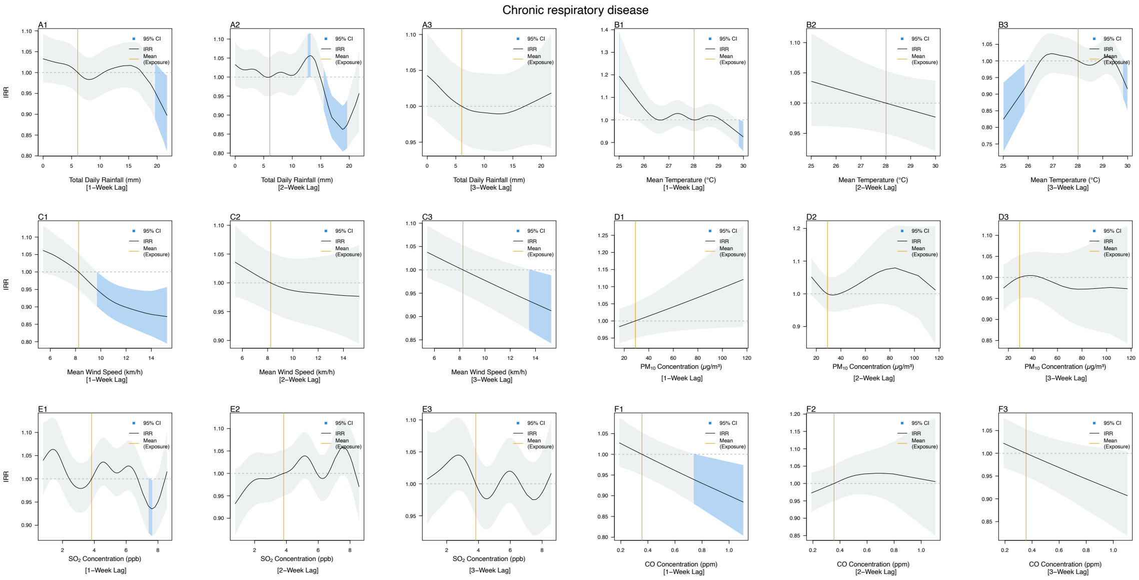
Chronic respiratory disease
A1
A2
A3
B1
B2
B3
C1
C2
C3
D1
D2
D3
E1
E2
E3
F1
F2
F3
95% CI
IRR
Mean (Exposure)
IRR
Total Daily Rainfall (mm) [1–Week Lag]
Total Daily Rainfall (mm) [2–Week Lag]
Total Daily Rainfall (mm) [3–Week Lag]
Mean Temperature (°C) [1–Week Lag]
Mean Temperature (°C) [2–Week Lag]
Mean Temperature (°C) [3–Week Lag]
Mean Wind Speed (km/h) [1–Week Lag]
Mean Wind Speed (km/h) [2–Week Lag]
Mean Wind Speed (km/h) [3–Week Lag]
PM_{10} Concentration (μg/m³) [1–Week Lag]
PM_{10} Concentration (μg/m³) [2–Week Lag]
PM_{10} Concentration (μg/m³) [3–Week Lag]
SO_2 Concentration (ppb) [1–Week Lag]
SO_2 Concentration (ppb) [2–Week Lag]
SO_2 Concentration (ppb) [3–Week Lag]
CO Concentration (ppm) [1–Week Lag]
CO Concentration (ppm) [2–Week Lag]
CO Concentration (ppm) [3–Week Lag]

Diabetes mellitus

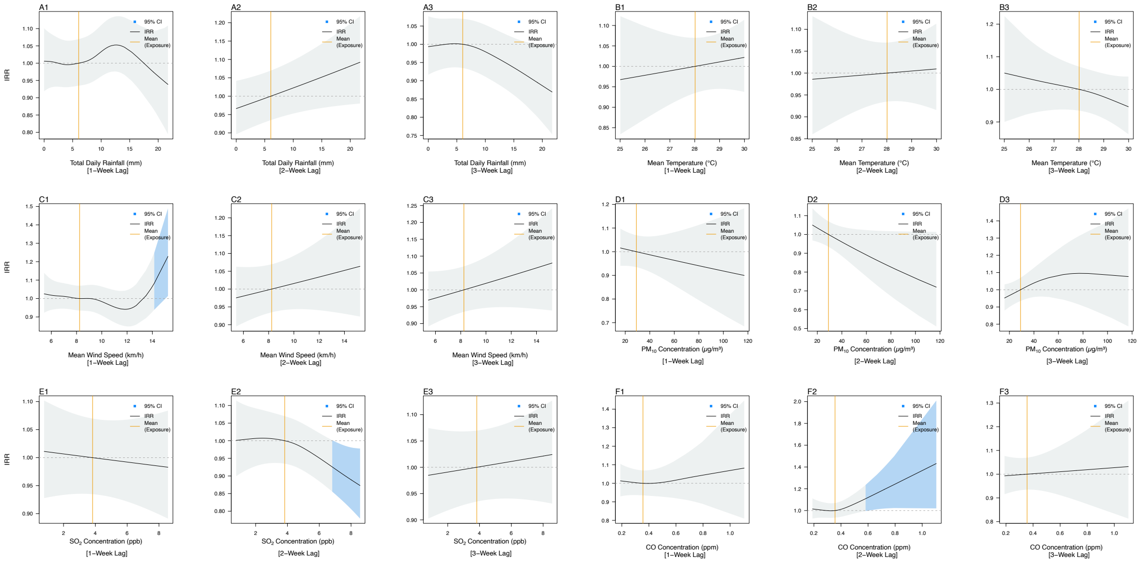

Digestive disease

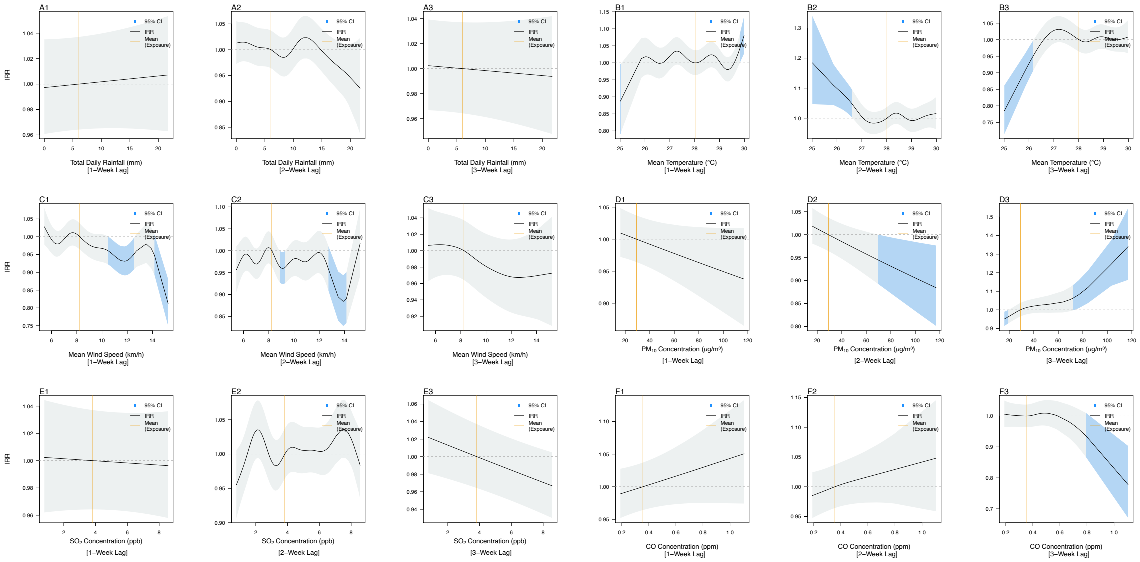

Endocrine disorders

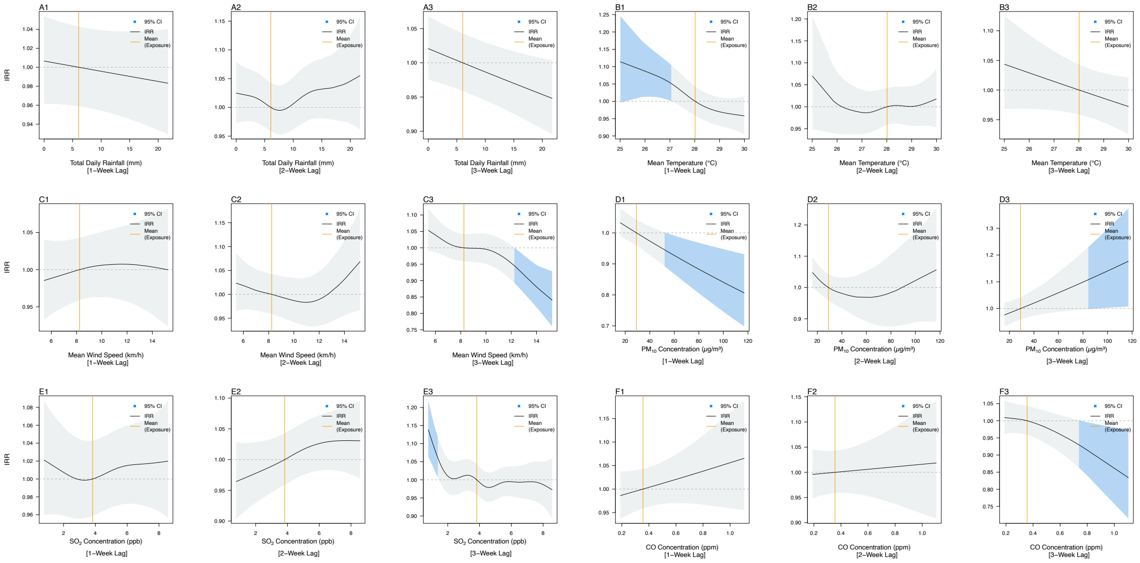

Genitourinary disorders

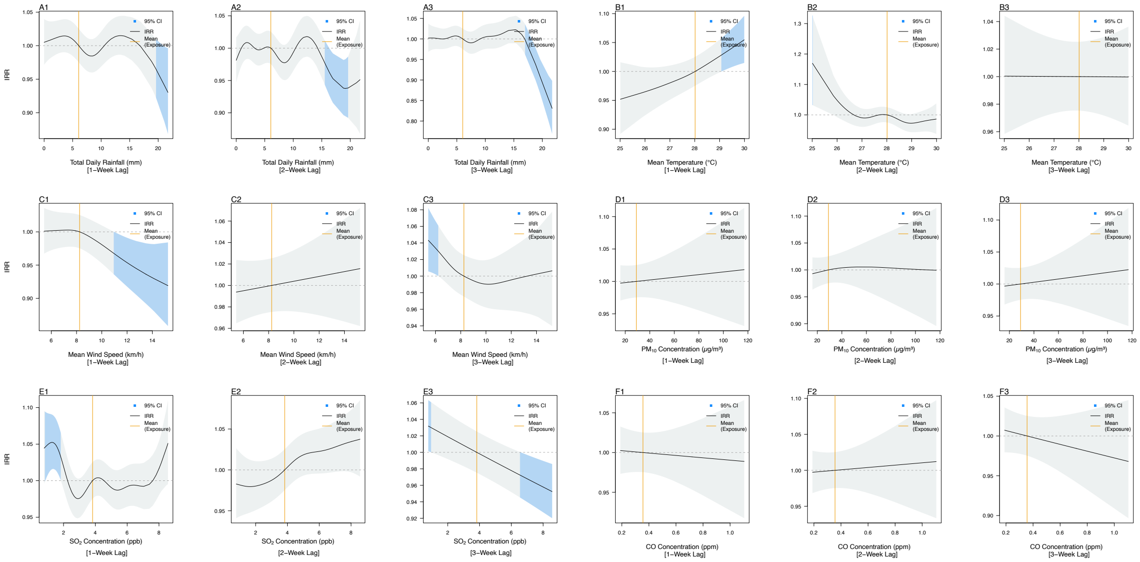

Infectious and Parasitic Diseases

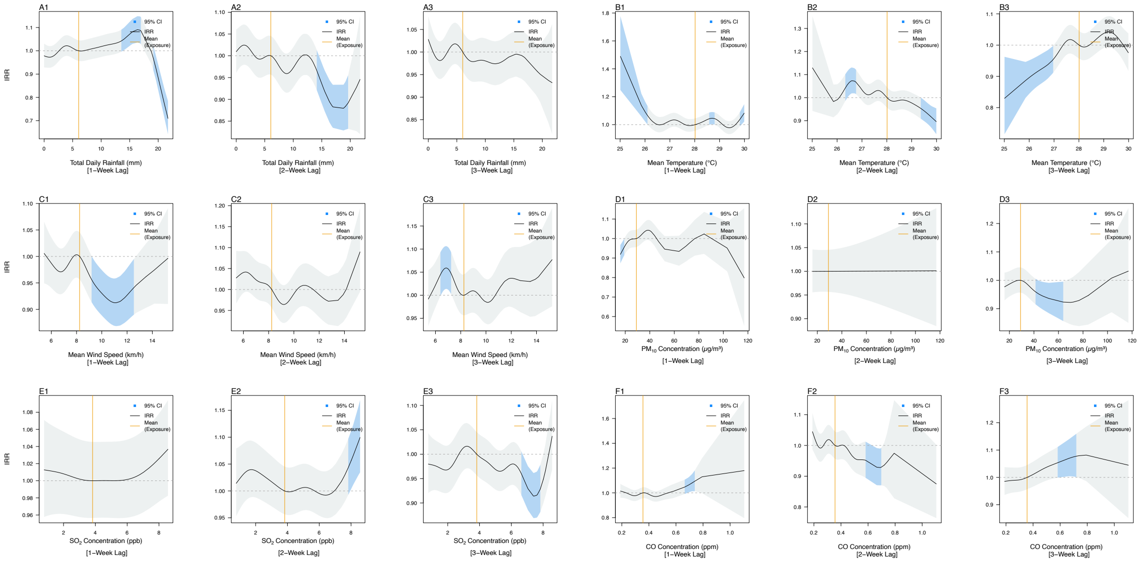

Musculoskeletal disease

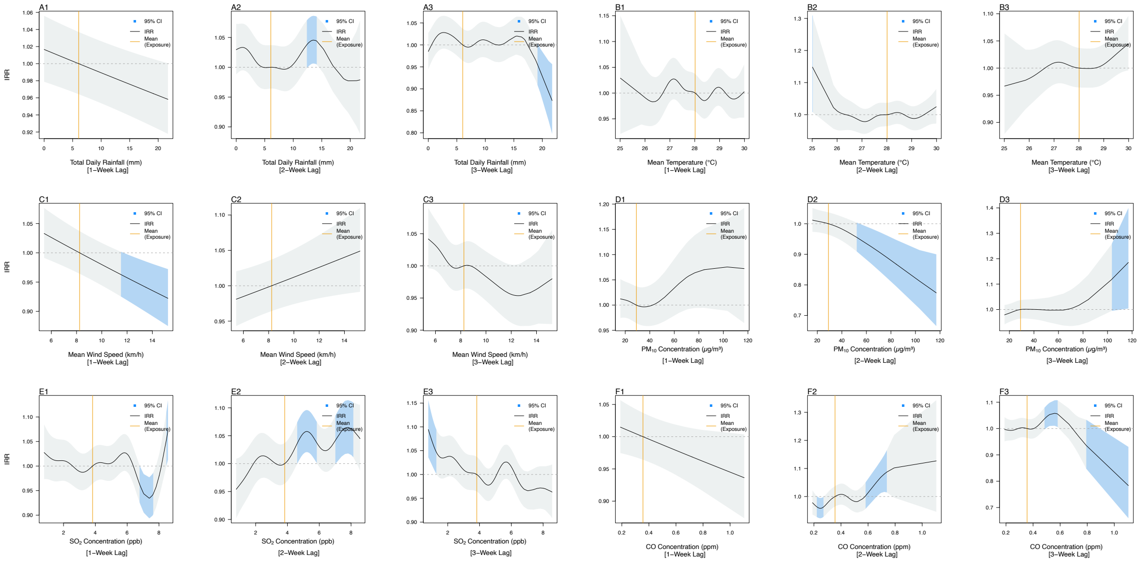

Neurological and sense disorders

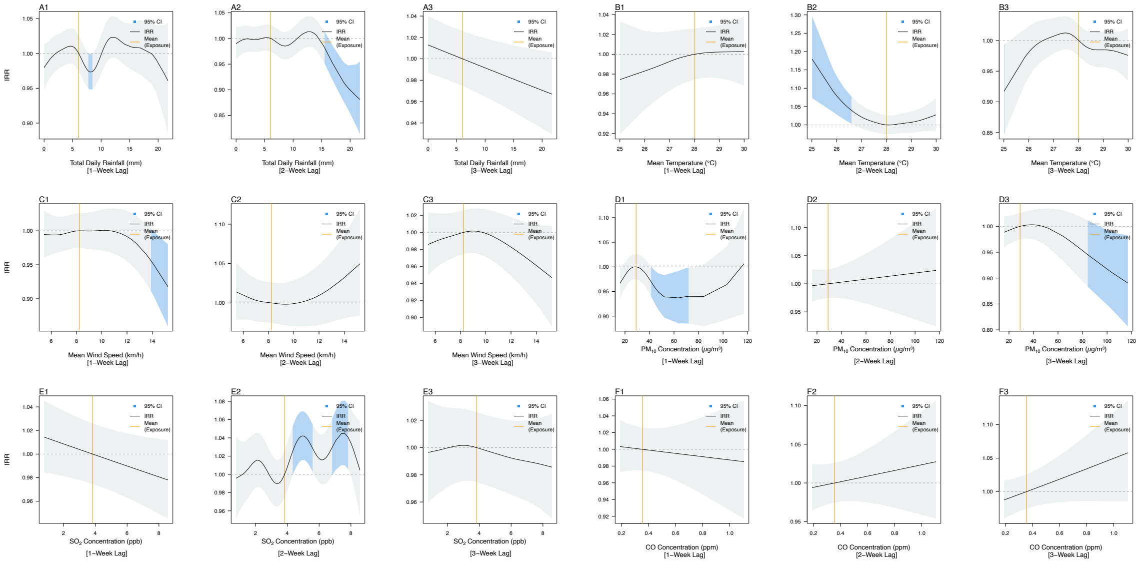

Oral Diseases

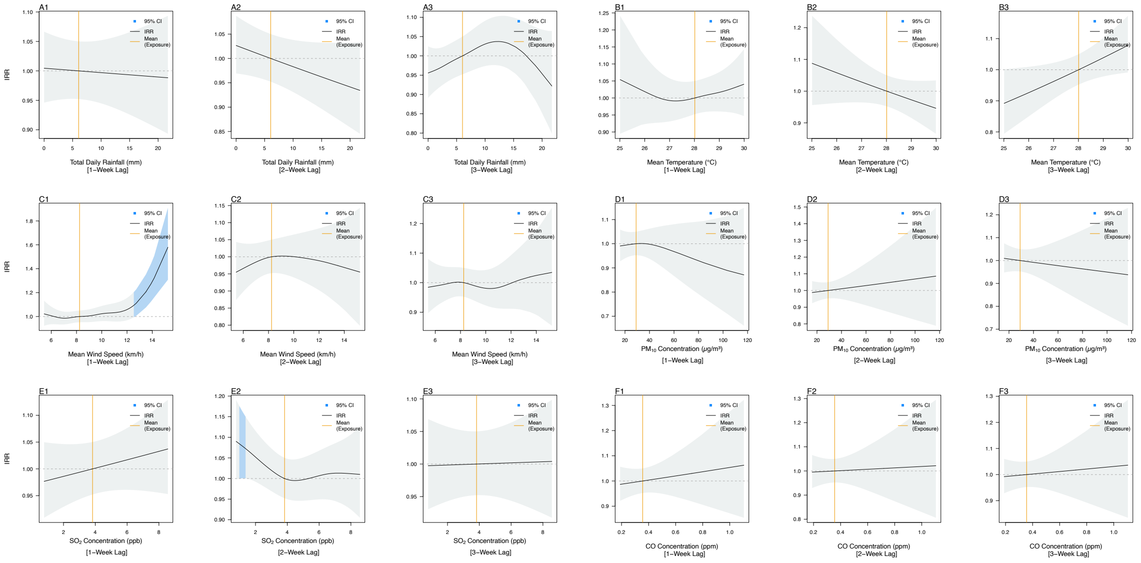

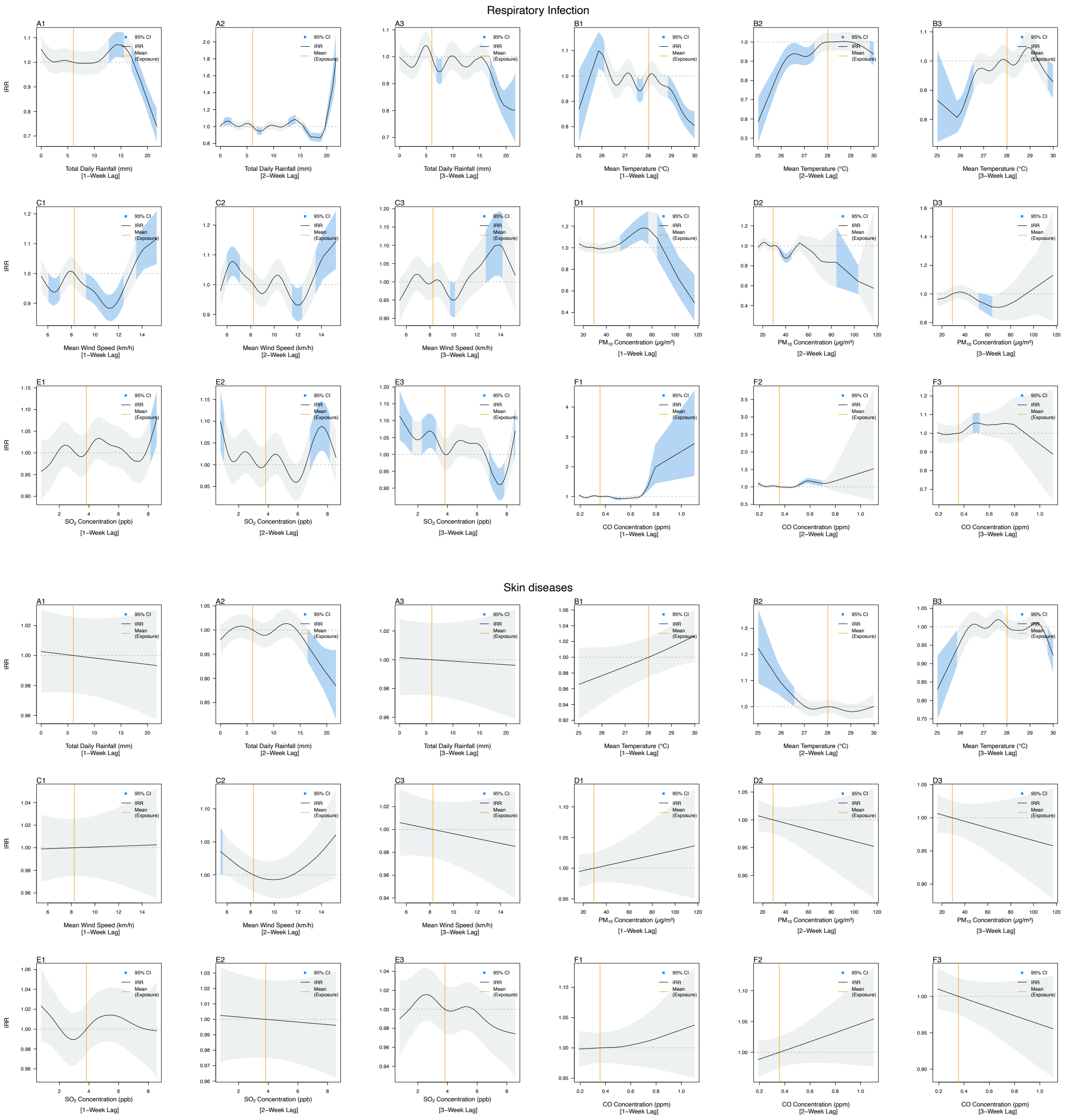

Figure S4. Incidence Rate Ratios of (A1–A3) Total Daily Rainfall (mm), (B1–B3) Mean Temperature (ºC), (C1–C3) Mean Wind Speed (km/h), (D1–D3) PM_{10} Concentration (μg/m^3), (E1–E3) SO_2 Concentration (ppb), (F1–F3) CO Concentration (ppm) for cause-specific ED admissions, at 1,2, and 3-week lags considered. Dark blue shaded areas represent IRRs where 95% confidence intervals do not cross 1, while the orange lines represent the mean recorded measurement of the respective exposure, as a reference value. Black lines represent IRR estimates, indicating the factor change in cause-specific ED admissions across the observed range of the exposure of interest, relative to the mean value of that exposure.

Cardiovascular Disease

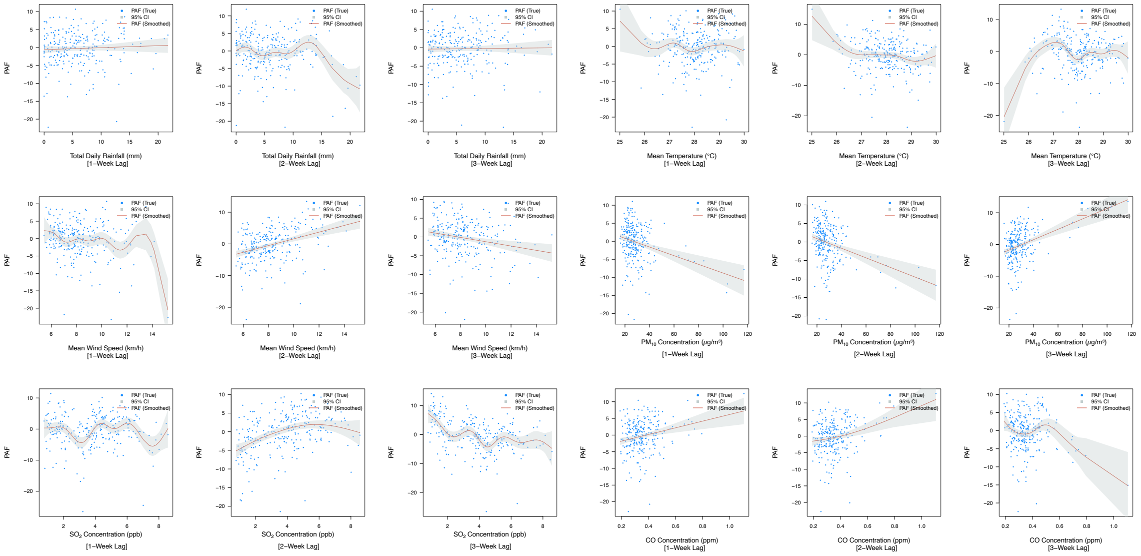

Chronic Respiratory Disease

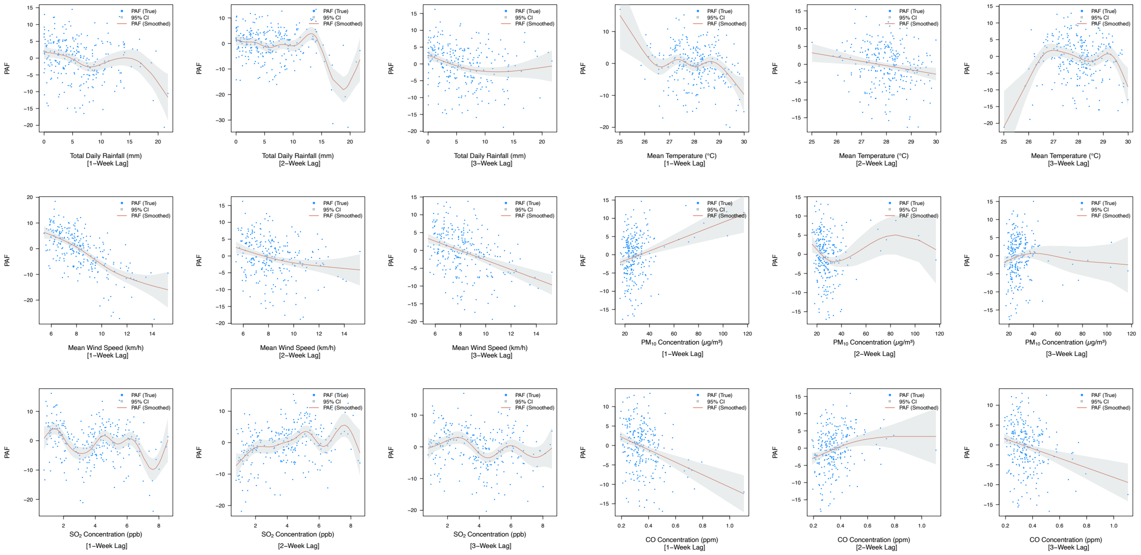

Diabetes Mellitus

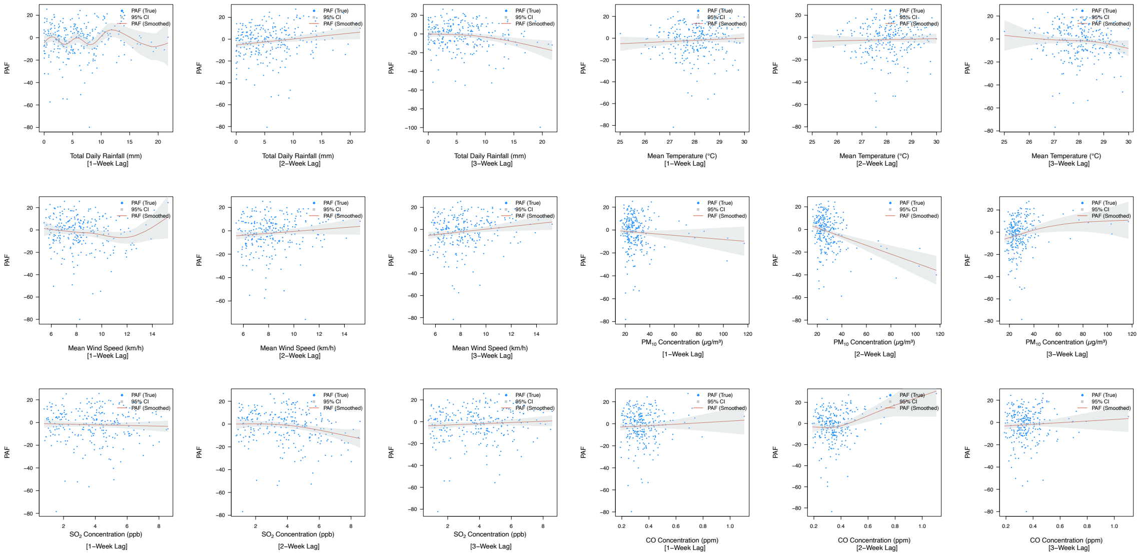

Digestive Disease

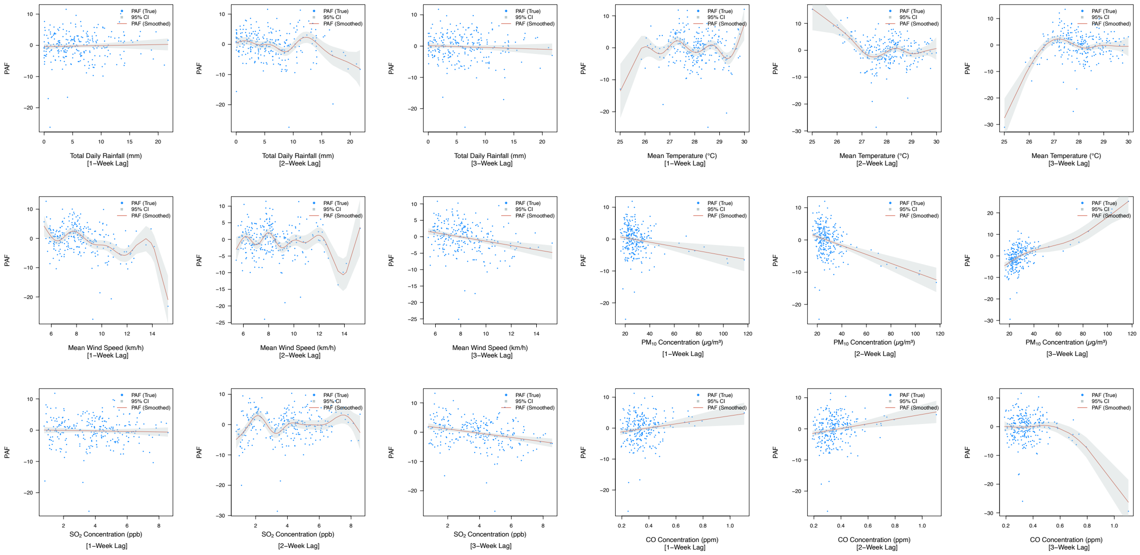

Endocrine Disorders

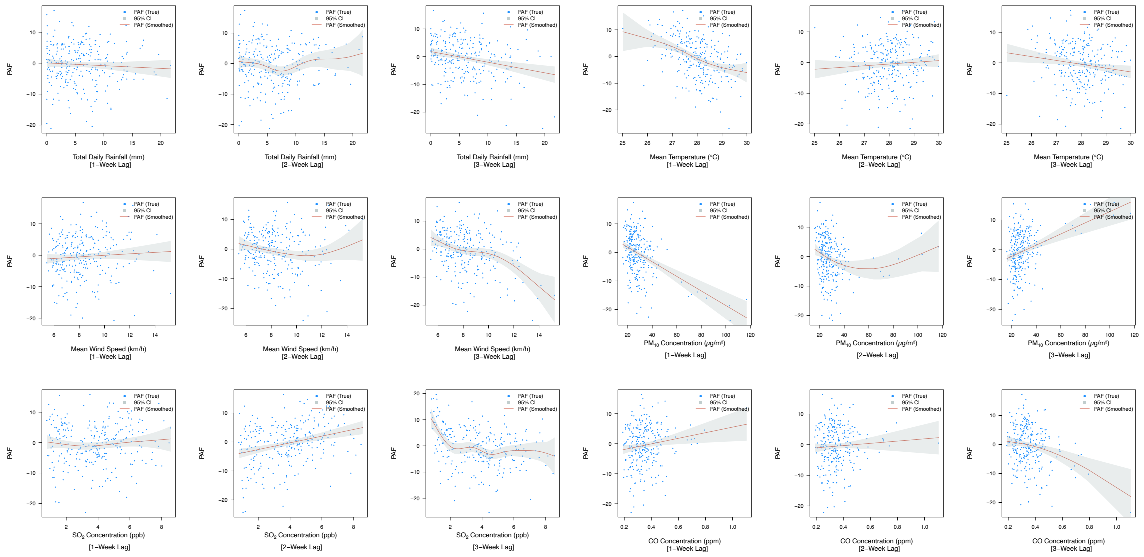

Genitourinary Disorders

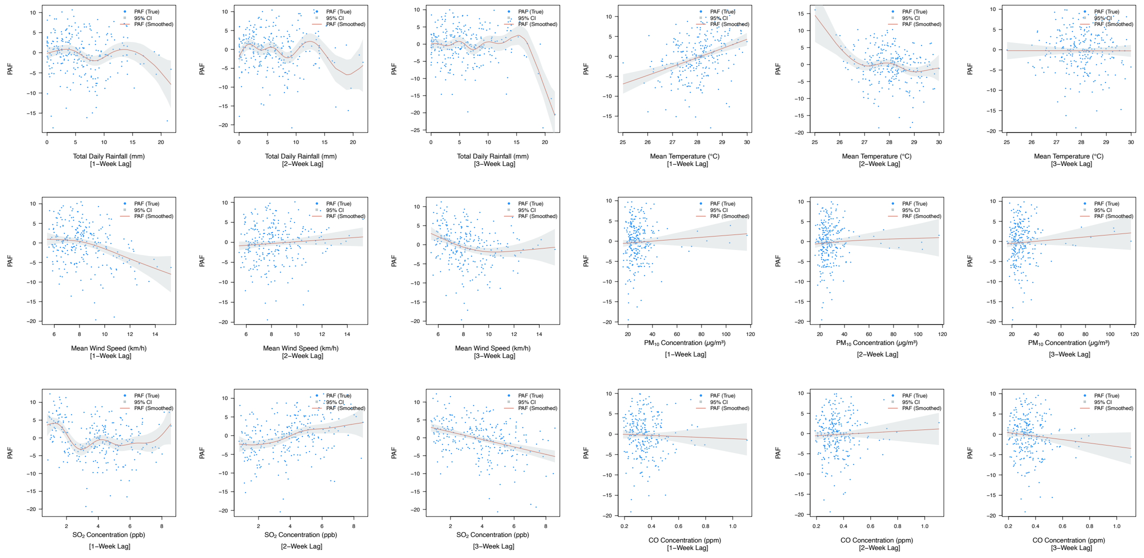

Infectious and Parasitic Diseases

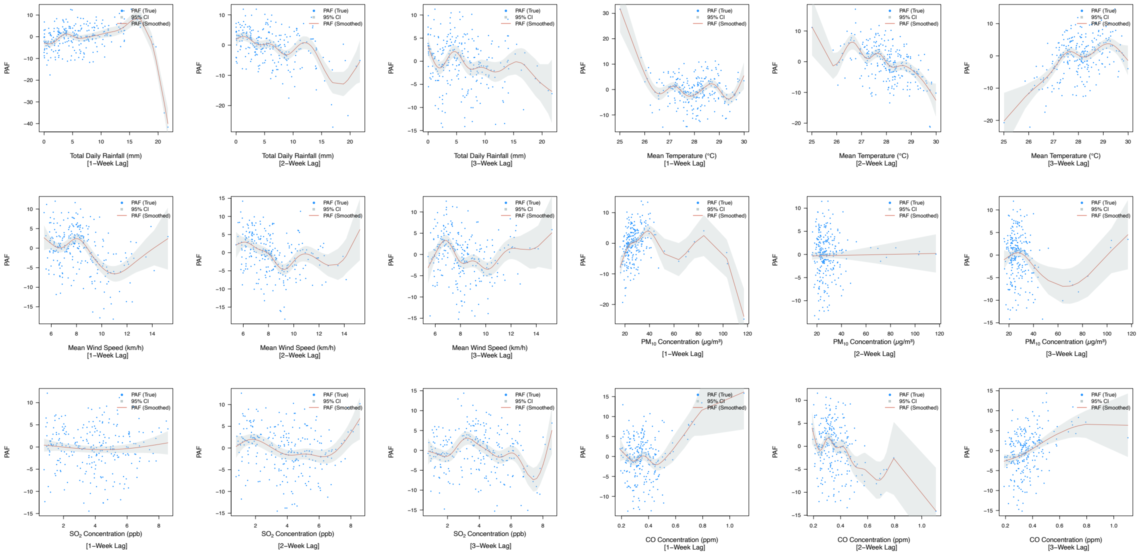

Musculoskeletal Disease

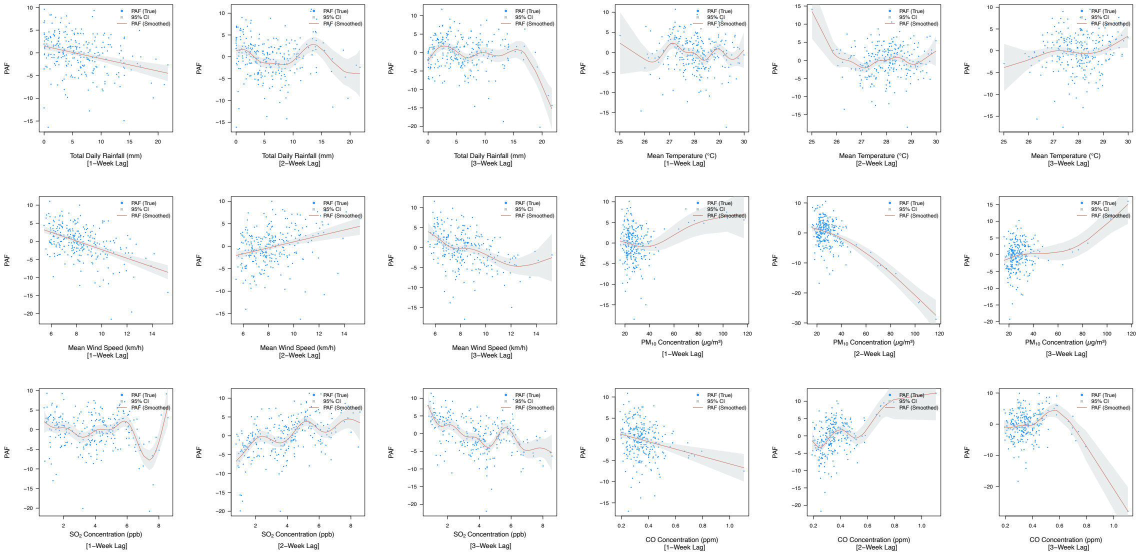

Neurological and Sense Disorders

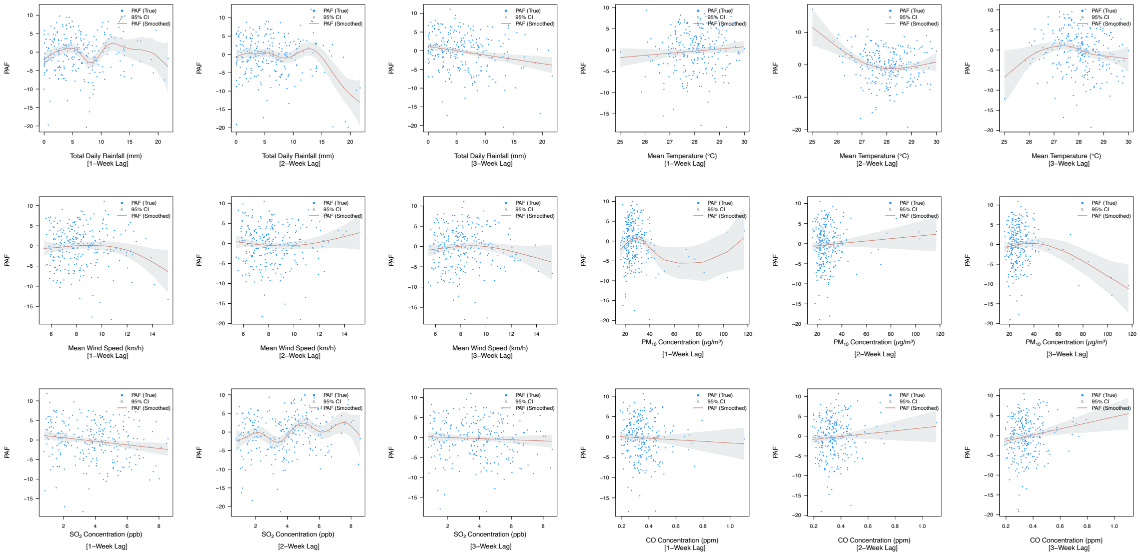

Oral Diseases

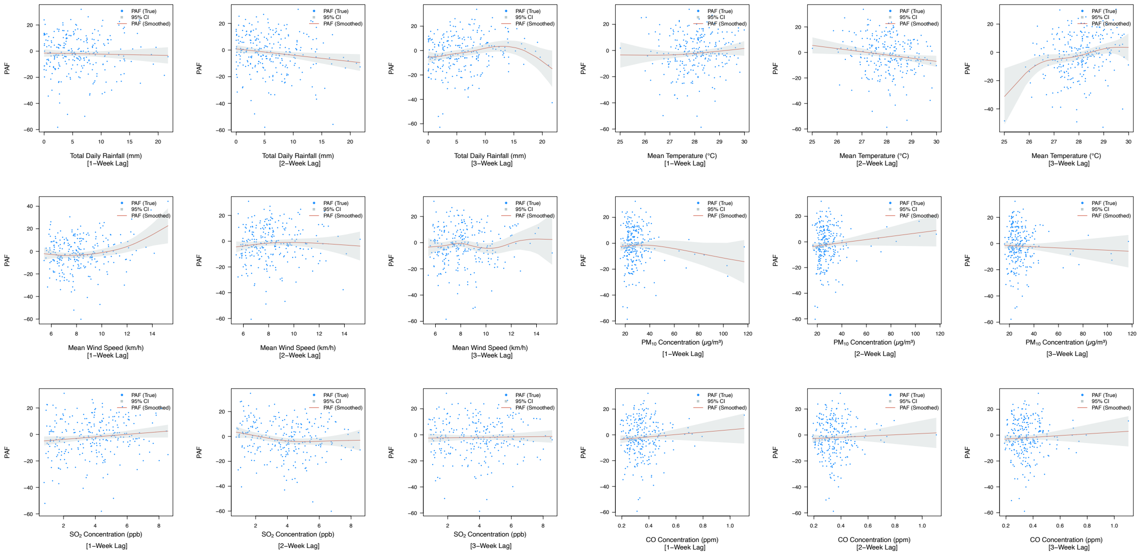

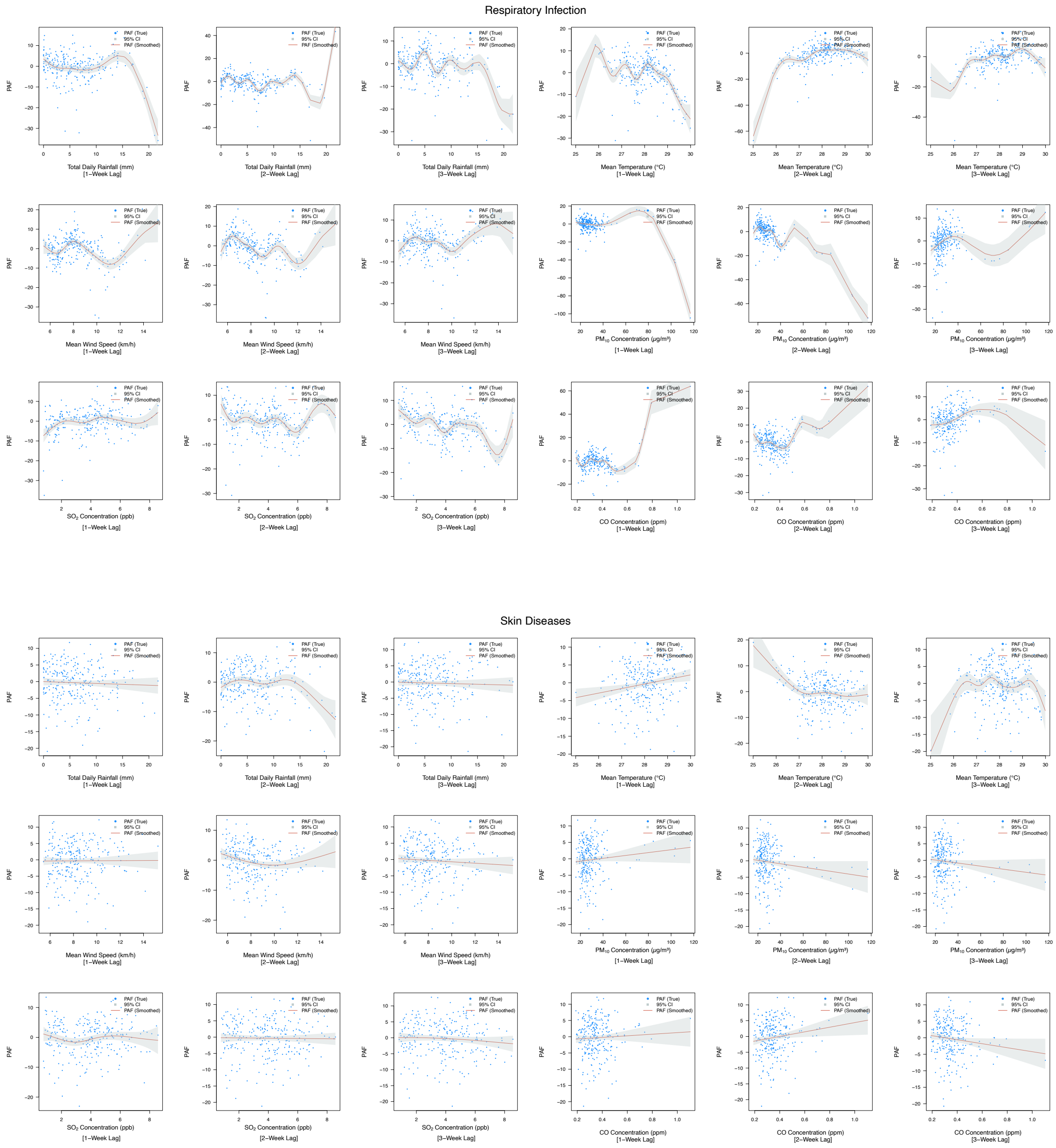

Figure S5. Population Attributable Fractions for Total Daily Rainfall (mm), Mean Temperature (ºC), Mean Wind Speed (km/h), PM_{10} Concentration (µg/m^3), (E1–E3) SO_2 Concentration (ppb), CO Concentration (ppm) at 1,2, and 3-week lags for all ED admission categories considered. The smoothed red lines represent the trend observed from the PAFs, while the blue dots represent the individual PAF values across the entire range of observed values, for each respective exposure.

	No Immediate Effects			*With Immediate Effects*					
	**Poisson**			**Quasi-Poisson**			**Negative Binomial**		
**Cardiovascular Disease**	**AIC**	**MAPE (%)**	**RMSE**	**AIC**	**MAPE (%)**	**RMSE**	**AIC**	**MAPE (%)**	**RMSE**
No lag	NA	NA	NA	NA	5.16	57.98	2905.4	5.15	57.93
1-Week Lag	3125.49	4.56	50.82	NA	4.85	53.96	2875.22	4.84	53.8
2-Week Lag	3048.80	4.24	46.80	NA	4.79	53.21	2875.02	4.73	52.54
3-Week Lag	**2900.24**	**3.59**	**39.95**	NA	4.35	48.39	2846.43	4.21	47.02
**Chronic Respiratory Disease**	**AIC**	**MAPE (%)**	**RMSE**	**AIC**	**MAPE (%)**	**RMSE**	**AIC**	**MAPE (%)**	**RMSE**
No lag	NA	NA	NA	NA	7.82	60.29	2918.67	7.81	60.25
1-Week Lag	3037.15	5.73	42.8	NA	6.15	45.74	2768.44	6.16	45.82
2-Week Lag	2920.65	5.16	37.79	NA	6.01	44.38	2762.15	6.00	44.35
3-Week Lag	**2824.20**	**4.53**	**33.19**	NA	5.78	42.70	2757.15	5.69	42.18
**Diabetes Mellitus**	**AIC**	**MAPE (%)**	**RMSE**	**AIC**	**MAPE (%)**	**RMSE**	**AIC**	**MAPE (%)**	**RMSE**
No lag	NA	NA	NA	NA	12.12	9.56	1954.20	12.08	9.55
1-Week Lag	1954.99	11.68	9.27	NA	11.88	9.25	1946.10	11.78	9.17
2-Week Lag	1933.27	11.05	8.74	NA	11.05	8.66	1927.93	10.91	8.56
3-Week Lag	1927.71	10.77	8.52	NA	10.75	8.47	**1925.28**	**10.42**	**8.24**
**Digestive Disease**	**AIC**	**MAPE (%)**	**RMSE**	**AIC**	**MAPE (%)**	**RMSE**	**AIC**	**MAPE (%)**	**RMSE**
No lag	NA	NA	NA	NA	7.99	104.80	3256.21	7.99	106.88
1-Week Lag	3341.98	4.41	61.81	NA	4.78	66.43	3001.95	4.77	66.41
2-Week Lag	3118.25	3.61	50.65	NA	4.65	65.03	2994.85	4.66	65.21
3-Week Lag	**2975.3**	**3.19**	**44.4**	NA	4.29	59.29	2965.68	4.12	57.42
**Endocrine Disorders**	**AIC**	**MAPE (%)**	**RMSE**	**AIC**	**MAPE (%)**	**RMSE**	**AIC**	**MAPE (%)**	**RMSE**
No lag	NA	NA	NA	NA	7.36	30.09	2523.94	7.36	30.15
1-Week Lag	2520.9	6.01	24.97	NA	5.98	24.6	2453.38	5.96	24.56
2-Week Lag	2502.6	5.82	24.09	NA	5.78	23.66	2443.18	5.71	23.52
3-Week Lag	**2454.47**	**5.19**	**21.48**	NA	5.55	22.73	2428.68	5.39	22.15
**Genitourinary Disorders**	**AIC**	**MAPE (%)**	**RMSE**	**AIC**	**MAPE (%)**	**RMSE**	**AIC**	**MAPE (%)**	**RMSE**
No lag	NA	NA	NA	NA	4.45	39.23	2685.62	4.45	39.2
1-Week Lag	2829.07	4.33	39.62	NA	4.19	37.49	2671.57	4.18	37.44
2-Week Lag	2801.98	4.10	37.48	NA	4.09	36.53	2668.92	4.06	36.30
3-Week Lag	**2712.46**	**3.68**	**32.91**	NA	3.78	34.40	2652.06	3.68	33.40
**Infectious and Parasitic Diseases**	**AIC**	**MAPE (%)**	**RMSE**	**AIC**	**MAPE (%)**	**RMSE**	**AIC**	**MAPE (%)**	**RMSE**
No lag	NA	NA	NA	NA	10.18	170.26	3462.40	10.17	170.58
1-Week Lag	4149.76	5.42	93.22	NA	5.84	103.78	3198.27	5.84	104.11

2-Week Lag	3679.29	4.41	76.5	NA	5.66	98.37	3183.60	5.61	97.87
3-Week Lag	**3390.43**	**3.55**	**62.45**	NA	5.52	95.68	3170.53	5.42	94.77
**Musculoskeletal Disease**	**AIC**	**MAPE (%)**	**RMSE**	**AIC**	**MAPE (%)**	**RMSE**	**AIC**	**MAPE (%)**	**RMSE**
No lag	NA	NA	NA	NA	5.01	76.36	3052.66	5.00	76.17
1-Week Lag	3324.69	4.18	64.61	NA	4.33	68.45	2989.46	4.30	67.9
2-Week Lag	3215.61	3.85	59.48	NA	4.21	65.86	2978.36	4.17	65.27
3-Week Lag	**3013.09**	**2.99**	**46.53**	NA	4.03	62.12	2957.57	3.97	61.54
**Neurological and Sense Disorders**	**AIC**	**MAPE (%)**	**RMSE**	**AIC**	**MAPE (%)**	**RMSE**	**AIC**	**MAPE (%)**	**RMSE**
No lag	NA	NA	NA	NA	4.73	48.03	2799.69	4.7	47.84
1-Week Lag	2908.03	4.39	44.07	NA	4.41	45.01	2771.7	4.39	44.89
2-Week Lag	2823.30	3.82	38.04	NA	4.39	44.29	2767.17	4.25	42.73
3-Week Lag	**2817.85**	**3.76**	**37.52**	NA	4.32	43.58	2764.01	4.28	43.31
**Oral Diseases**	**AIC**	**MAPE (%)**	**RMSE**	**AIC**	**MAPE (%)**	**RMSE**	**AIC**	**MAPE (%)**	**RMSE**
No lag	NA	NA	NA	NA	12.13	9.93	1969.29	12.08	9.89
1-Week Lag	1960.46	11.63	9.48	NA	11.52	9.33	1951.34	11.47	9.28
2-Week Lag	1954.45	11.24	9.17	NA	11.25	9.20	1952.47	10.95	8.96
3-Week Lag	**1953.27**	**11.03**	**8.99**	NA	11.34	9.18	1953.71	11.24	9.09
**Respiratory Infection**	**AIC**	**MAPE (%)**	**RMSE**	**AIC**	**MAPE (%)**	**RMSE**	**AIC**	**MAPE (%)**	**RMSE**
No lag	NA	NA	NA	NA	13.42	371.53	3879.92	13.54	376.80
1-Week Lag	6552.71	6.42	179.69	NA	6.93	200.16	3564.74	6.95	201.67
2-Week Lag	5477.22	5.32	148.98	NA	6.86	195.33	3557.59	6.90	197.71
3-Week Lag	**4395.21**	**3.99**	**112.91**	NA	6.74	188.96	3547.37	6.73	190.31
**Skin Diseases**	**AIC**	**MAPE (%)**	**RMSE**	**AIC**	**MAPE (%)**	**RMSE**	**AIC**	**MAPE (%)**	**RMSE**
No lag	NA	NA	NA	NA	5.93	53.94	2861.65	5.91	53.85
1-Week Lag	3010.61	4.87	44.46	NA	5.22	47.44	2802.26	5.20	47.24
2-Week Lag	2937.88	4.51	41.36	NA	4.87	44.49	2781.41	4.84	44.14
3-Week Lag	**2865.59**	**4.28**	**39.29**	NA	4.75	43.42	2770.88	4.70	43.00

Table S1. Sensitivity Analysis for models considered. Models belonging to the 'No Immediate Effects' column are models that do not have any immediate exposures specified in their regression equations (i.e. they only contain lagged exposures), while models belonging to the "With Immediate Effects" columns contain both lagged and immediate exposures. Models with best-fit are underlined.
